# Characterization of two distinct immortalized endothelial cell lines, EA.hy926 and HMEC-1, for in vitro studies: exploring the impact of calcium electroporation, Ca^2+^ signaling and transcriptomic profiles

**DOI:** 10.1186/s12964-024-01503-2

**Published:** 2024-02-12

**Authors:** Barbara Lisec, Tim Bozic, Iva Santek, Bostjan Markelc, Milka Vrecl, Robert Frangez, Maja Cemazar

**Affiliations:** 1 Department of Experimental Oncology, Institute of Oncology Ljubljana, Zaloska cesta 2, SI-1000 Ljubljana, Slovenia; 2 Faculty of Medicine, University of Ljubljana, Vrazov trg 2, SI-1000 Ljubljana, Slovenia; 3 Institute of Preclinical Sciences, Veterinary Faculty, University of Ljubljana, Gerbiceva 60, SI-1000 Ljubljana, Slovenia; 4 Faculty of Health Sciences, University of Primorska, Polje 42, SI-6310 Izola, Slovenia

**Keywords:** Endothelial cells, Calcium electroporation, Calcium kinetics, Transcriptomic profiling

## Abstract

**Background:**

Disruption of Ca^2+^ homeostasis after calcium electroporation (CaEP) in tumors has been shown to elicit an enhanced antitumor effect with varying impacts on healthy tissue, such as endothelium. Therefore, our study aimed to determine differences in Ca^2+^ kinetics and gene expression involved in the regulation of Ca^2+ ^signaling and homeostasis, as well as effects of CaEP on cytoskeleton and adherens junctions of the established endothelial cell lines EA.hy926 and HMEC-1.

**Methods:**

CaEP was performed on EA.hy926 and HMEC-1 cells with increasing Ca^2+^ concentrations. Viability after CaEP was assessed using Presto Blue, while the effect on cytoskeleton and adherens junctions was evaluated via immunofluorescence staining (F-actin, α-tubulin, VE-cadherin). Differences in intracellular Ca^2+^ regulation ([Ca^2+^]_i_) were determined with spectrofluorometric measurements using Fura-2-AM, exposing cells to DPBS, ionomycin, thapsigargin, ATP, bradykinin, angiotensin II, acetylcholine, LaCl_3_, and GdCl_3_. Molecular distinctions were identified by analyzing differentially expressed genes and pathways related to the cytoskeleton and Ca^2+^ signaling through RNA sequencing.

**Results:**

EA.hy926 cells, at increasing Ca^2+^ concentrations, displayed higher CaEP susceptibility and lower survival than HMEC-1. Immunofluorescence confirmed CaEP-induced, time- and Ca^2+^-dependent morphological changes in EA.hy926’s actin filaments, microtubules, and cell–cell junctions. Spectrofluorometric Ca^2+^ kinetics showed higher amplitudes in Ca^2+^ responses in EA.hy926 exposed to buffer, G protein coupled receptor agonists, bradykinin, and angiotensin II compared to HMEC-1. HMEC-1 exhibited significantly higher [Ca^2+^]_i_ changes after ionomycin exposure, while responses to thapsigargin, ATP, and acetylcholine were similar in both cell lines. ATP without extracellular Ca^2+^ ions induced a significantly higher [Ca^2+^]_i_ rise in EA.hy926, suggesting purinergic ionotropic P2X and metabotropic P2Y receptor activation. RNA-sequencing analysis showed significant differences in cytoskeleton- and Ca^2+^-related gene expression, highlighting upregulation of *ORAI2*, *TRPC1*, *TRPM2*, *CNGA3*, *TRPM6*, and downregulation of *TRPV4* and *TRPC4* in EA.hy926 versus HMEC-1. Moreover, KEGG analysis showed upregulated Ca^2+^ import and downregulated export genes in EA.hy926.

**Conclusions:**

Our finding show that significant differences in CaEP response and [Ca^2+^]_i_ regulation exist between EA.hy926 and HMEC-1, which may be attributed to distinct transcriptomic profiles. EA.hy926, compared to HMEC-1, displayed higher susceptibility and sensitivity to [Ca^2+^]_i_ changes, which may be linked to overexpression of Ca^2+^-related genes and an inability to mitigate changes in [Ca^2+^]_i_. The study offers a bioinformatic basis for selecting EC models based on research objectives.

**Supplementary Information:**

The online version contains supplementary material available at 10.1186/s12964-024-01503-2.

## Background

The vascular endothelium is composed of an endothelial cell (EC) monolayer and has many important functions, including regulation of immune responses, control of blood coagulation states, permeability of molecules between blood and tissue, angiogenesis, vessel repair, and vascular tone [1]. These diverse functions confer on ECs indispensable roles in the body’s normal homeostasis and, in the case of abnormal function, in many pathological conditions [2]. Several endothelial cell lines have been described for the study of vascular endothelium. The most commonly used cells are primary human umbilical vein endothelial cells (HUVECs) [3]. Primary HUVECs retain EC properties, including signaling pathways and the expression of specific surface markers [4]. Although HUVECs are available as a mixture from multiple donors long-term in vitro experiments pose challenges due to inherent inter-donor variability, limited proliferative capacity and potential phenotypic drift [5]. To standardize experimental conditions and obtain reproducible results, many immortalized endothelial cell lines have been developed, such as the macrovascular and microvascular endothelial cell lines EA.hy926 and HMEC-1, respectively. Whereas EA.hy926 is a hybrid cell line derived from the fusion of human umbilical vein ECs with the permanent human adenocarcinoma epithelial cell line A549 [6], the HMEC-1 cell line was developed by transfecting human dermal microvascular endothelial cells (HMECs) with the SV40 large T antigen [7]. The selection of the most suitable EC type for a successful experimental design is of great importance because ECs are heterogeneous in their functions, which strongly depend on tissue origin and is reflected in their specific gene expression profile [8].

To sustain endothelial homeostasis and execute needed functions, ECs depend to varying degrees on changes in intracellular free Ca^2+^ concentration ([Ca^2+^]_i_) [9]. Calcium is a ubiquitous second messenger that is tightly regulated inside the cell by Ca^2+^ ion channels and receptors [10, 11]. An increase in [Ca^2+^]_i_ in a cell triggers a variety of cellular processes, including proliferation, migration, and cell death [12, 13]. Excessive influx and uptake of Ca^2+^ in intracellular stores, such as the endoplasmic reticulum (ER) and mitochondria, signifies cell stress and can lead to irreversible Ca^2+^ overload, resulting in cell death triggered by mitochondrial dysfunction and subsequent energy production failure [14–16]. Therefore, Ca^2+^ has been investigated as an antitumor treatment in combination with electroporation (EP) as a nonmutagenic and inexpensive alternative to electrochemotherapy (ECT), which can induce tumor necrosis via ATP depletion [17–19]. In preclinical studies, calcium electroporation (CaEP) with local intratumoral administration of a Ca^2+^ solution showed dose-dependent antitumor efficacy, with the number of tumors cured depending on tumor type [20–22]. However, when the same tumor type was treated in immunocompromised mice, a similar effect was not observed, indicating that an immune response mediated by nontumor cells was involved [21]. In addition, observations from these studies suggest that CaEP acts primarily on tumor cells, while surrounding cells, such as muscle and ECs, remain largely unaffected. This has set in motion a series of studies focusing on the importance of nontumor cells in tumor treatment with CaEP-based protocols [23, 24]. In vitro, significantly less cell death was observed in normal muscle cells compared to cancer muscle cells subjected to CaEP both at low calcium (0.5 mM) and high calcium (5 mM calcium) concentrations for most of the EP parameters tested [23]. Furthermore, differences in the response to CaEP were also observed between nontumor cells, which suggests that susceptibility to CaEP is strongly dependent on the expression of proteins involved in the regulation of Ca^2+^ signaling, [Ca^2+^]_i_ homeostasis [11, 25] and the cytoskeleton [26]. Thus, understanding Ca^2+^-related gene expression profiles that determine the effects of CaEP on normal tissues is of paramount importance not only for optimizing the treatment method but also for selecting the most appropriate cell line model in Ca^2+^-related studies. Another important aspect that is often omitted in in vitro studies is the presence of physical forces on the endothelium that mediate biological responses, such as shear stress, cyclic stretch and stiffness [27]. Normally, ECs are cultured on stiff surfaces and in the absence of pressure and flow, which greatly affects the functionality of these cells in biological systems [28]. Because the mechanical forces induced by sustained blood flow also affect the Ca^2+^ kinetics of the vascular endothelium, it is crucial to understand Ca^2+^ dynamics under these conditions, which can be mimicked in microfluidic and kinetic assays [29–34].

Although cytotoxicity profiles of different normal cells after CaEP have been obtained, the human endothelial cell lines EA.hy926 and HMEC-1 have not yet been tested. The cytoskeleton of the vasculature endothelium has been extensively studied after EP but not to the same extent after CaEP [35–37]. Moreover, transcriptomic profiling of EA.hy926 and HMEC-1 cells with respect to Ca^2+^-related signaling would provide a deeper understanding of the underlying molecular background responsible for the observed responses of CaEP in normal tissue. Therefore, the aim of this study was to compare the two most widely used immortalized endothelial cell lines, EA.hy926 and HMEC-1, after CaEP. Specifically, we aimed to determine the i) direct dose-dependent cytotoxic effect of CaEP on EA.hy926 and HMEC-1 cells, ii) indirect effect of treatment on the cytoskeleton of ECs by immunofluorescence staining of actin filaments, microtubules and cell–cell junctions (VE-cadherin), iii) kinetic changes in [Ca^2+^]_i_ and iv) RNA transcriptomic profiles of untreated EA.hy926 and HMEC-1 cells by RNA sequencing (RNA-seq) to identify differentially expressed genes between the investigated cell lines and provide molecular cues, which may be responsible for their distinct responses to CaEP.

## Methods

### Endothelial cell cultures

EA.hy926 and HMEC-1 cells were purchased from American Type Culture Collection (ATCC) and routinely tested for mycoplasma using the MycoAlert™ Plus Mycoplasma Detection Kit (Lonza). EA.hy926 cells were cultured in Advanced DMEM medium (Gibco) supplemented with 5% FBS. HMEC-1 cells were cultured in MCDB 131 medium (Gibco) supplemented with 10% fetal bovine serum (FBS, Gibco), 10 ng/mL epidermal growth factor and 1 μg/mL hydrocortisone. All media were supplemented with 10 mM L-glutamine, 50 mg/mL gentamicin and 100 U/mL penicillin. Although these media formulations include the same amount of Ca^2+^ ions (CaCl_2_ 0.2 g/L), the percentage of FBS and other factors might have an impact on gene expression [38]. The authors adhered to the supplier’s guidelines to ensure optimal cell growth and maintain physiological relevance to the native cellular environment. This decision aimed to uphold consistency and accuracy in the experimental conditions. In experiments, cells in up to 10 passages were used. Cells were maintained at 37 °C in a humid atmosphere with 5% CO_2_ until they formed a confluent monolayer. Unless stated otherwise, a confluent monolayer of cells was washed with PBS, detached with trypsin and collected by centrifugation for further experiments.

### Reagents and antibodies

Electroporation buffer (EPB) contained 136 mM NaCl, 5 mM KCl, 2 mM MgCl_2_, 10 mM HEPES, and 10 mM glucose (pH 7.2, 300–310 mOsm/kg) [39]. For calcium electroporation, EPB was supplemented with increasing concentrations of CaCl_2_ (0.5 mM – 3 mM). All dilutions were prepared in EPB buffer. Cytoskeleton stabilizing buffer (CSB; pH 6.8) for immunofluorescence experiments contained 80 mM PIPES, 150 mM NaCl, 5 mM EGTA and 5 mM MgCl_2_ at final concentrations.

The primary antibodies used for immunofluorescence studies were goat anti-human VE-cadherin (#AF938; R&D Systems) and mouse anti-human α-tubulin (#A11126; Thermo Fischer Scientific). The secondary antibodies used were donkey anti-mouse IgG H&L (Alexa Fluor® 488) (#ab150105; Abcam) and donkey anti-goat IgG (H + L) (Alexa Fluor® 647 AffiniPure) (#705–605-147; Jackson ImmunoResearch). For actin filament staining, Alexa Fluor 546 phalloidin (#A22283; Thermo Fisher Scientific) was used.

Ethylene glycol, tetraacetic acid, ionomicine, lanthanum (III) chloride (LaCl_3_), bradykinin, angiotensin II, ATP, acetylcholine iodide and gadolinium (III) chloride (GdCl_3_) were obtained from Sigma–Aldrich. Thapsigargin (TG) and Fura-2/acetoxy-methyl ester (Fura-2-AM) were purchased from Thermo Fisher Scientific. For fluorometric kinetic measurements of intracellular free Ca^2+^ concentration ([Ca^2+^]_i_), cells were stained with Fura-2-AM (#F1201, Thermo Fisher Scientific) and treated with different substances: 10 μM ionomycin (#I24222, Thermo Fisher Scientific), 1 μM thapsigargin (#T7459, Thermo Fisher Scientific), 100 μM ATP (#FLAAS-1VL; Merck), 10 μM bradykinin (#B3259; Merck), 10 μM angiotensin II (#A9525, Merck), 300 μM acetylcholine (#A7000, Merck), 500 μM LaCl_3_ and 500 μM GdCl_3_. All substances were dissolved in Dulbecco’s phosphate-buffered saline (DPBS) to their final concentration prior to the kinetic experiments.

### Electroporation of EA.hy926 and HMEC-1 cells

#### Adherent cells

Cells were seeded onto 12-well removable chamber slides (Ibidi) coated with 0.5% gelatin (Sigma–Aldrich) at a density of 1 × 10^4^ cells/well for HMEC-1 cells or 1.5 × 10^4^ cells/well for EA.hy926 cells. Cells were cultured until a confluent monolayer was reached and then for another 3 days, so the cell junctions were properly formed (in total 7 days). We determined similar doubling time (approx. 24 h) of both cell lines (Supplementary Fig. S1). However, in the case of adherent experiments, the cell seeding density was adjusted due to the larger size of HMEC-1 cells compared to Ea.hy926 cells. This adjustment was made to ensure consistent cell confluence and monolayer formation at the time of the experiment for both cell lines.

For optimization of EP parameters, cells were washed with EPB buffer, followed by the addition of EPB buffer containing 100 μM propidium iodine (PI). Cells were electroporated with 8 square-wave electric pulses with an amplitude of 0 V, 100 V, 200 V, 300 V, 400 V, 500 V, 600 V, 700 V, 800 V or 900 V, pulse length 100 μs and repetition frequency 1 Hz. Slides of EA.hy926 and HMEC-1 monolayers after electroporation were stained to determine the cell morphology and permeabilization.

In the case of CaEP, cells were washed with EPB and then incubated in EPB or EPB with CaCl_2_ (0.5–3 mM) during electroporation. Pulse parameters were as follows: 8 square-wave electric pulses, amplitude to distance ratio 1000 V/cm, pulse length 100 μs, repetition frequency 1 Hz. Cells were incubated for 5 min before the EP buffer was changed to the cell culture medium. For both optimization of EP parameters and CaEP, pulses were generated by a Jouan GHT beta pulse generator (LEROY biotech) and delivered via plate electrodes spaced 6.45 mm apart.

#### Cells in suspension

The viability of EA.hy926 and HMEC-1 cells was assessed using cells in suspension. Cells were collected by trypsinization, washed in EPB, resuspended in EPB at a density of 25 × 10^6^ cells/mL and aliquoted. For optimization of EP parameters, cell aliquots were electroporated by applying pulses with increasing amplitude and the same parameters as for the adherent cells.

For CaEP, cell aliquots were resuspended in EPB with CaCl_2_ (final concentration 0.5–3 mM) and electroporated. Pulse parameters were as follows: 8 square-wave electric pulses, amplitude to distance ratio 1000 V/cm, pulse length 100 μs, repetition frequency 1 Hz. After a 5 min incubation in a 24-well low-attachment plate, complete cell culture medium was added to the cells, and then the cells were seeded at a density of 4000 cells/well in a 96-well plate. At different time points (4 h, 24 h or 72 h) after treatment, 10 μL Presto Blue reagent (Thermo Fischer Scientific) was added to the cells, and after 1 h incubation, their viability was measured with a Cytation 1 Cell Imaging Multi-Mode Reader (BioTek). For both the optimization of EP parameters and CaEP, pulses were generated by a Jouan GHT beta pulse generator (LEROY biotech) and delivered via plate electrodes spaced 2.40 mm apart.

#### Staining of EA.hy926 and HMEC-1 monolayers

To determine the morphology of EA.hy926 and HMEC-1 cells, slides with monolayers were air-dried for 30 min after electroporation. Then, the slides were fixed with cooled 100% methanol for 7 min, washed with H_2_O, and incubated with Giemsa stain for 10 min. The stained slides were air-dried for 1 h, mounted with ProLong™ Glass Antifade Mountant (Thermo Fischer Scientific) and covered with a coverslip. Bright-field images of the stained slides were obtained with a Zeiss LSM 800 confocal microscope (Carl Zeiss) using a 20x objective (NA 0.8) and an Axiocam 506 digital camera.

**Fig. 1:**
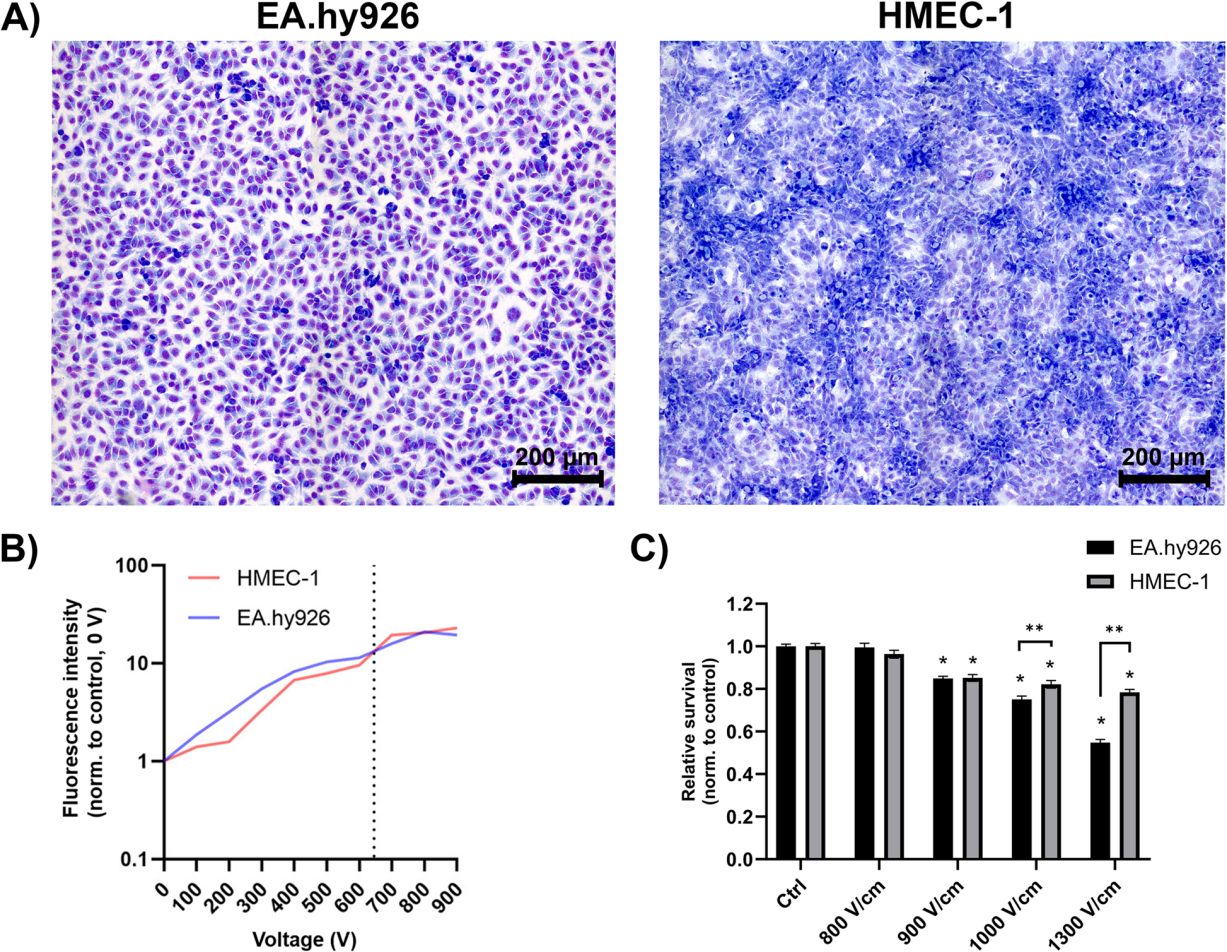
Endothelial cells EA.hy926 and HMEC-1 after electroporation. **A** Untreated monolayers of EA.hy926 and HMEC-1 cells stained with Giemsa stain. Scale bar: 200 μm. **B** Permeabilization of ECs after exposure to electric pulses with increasing voltage. Fluorescence intensity of propidium iodine (PI) normalized to the control (0 V) 1 h after electroporation of EA.hy926 and HMEC-1 cells. The dotted vertical line marks the voltage of 645 V (1000 V/cm), which results in equivalent permeabilization of both cell lines. **C** Cell survival of ECs after exposure to electric pulses with increasing voltage. Normalized cell survival of EA.hy926 and HMEC-1 cells in suspension 72 h after exposure to electric pulses with increasing electric field. The values are presented as AM ± SE (*n* ≥ 40). Statistical significance was determined by a t-test. A *P* value of < 0.05 was considered to be statistically significant (*P < 0.05 vs control (Ctrl) (0 V), **P < 0.05 between EA.hy926 and HMEC-1 cells)

To determine the permeabilization of EA.hy926 and HMEC-1 cells after electroporation using PI, slides with monolayers were first fixed with 4% PFA and incubated for 10 minutes at 37 °C. The fixed cells were washed three times with PBS and stained with Hoechst 33342 solution (3 μg/mL; Thermo Fisher Scientific) for 10 minutes at room temperature, followed by washing three times with PBS for 5 minutes. Excess liquid and silicone chambers were removed, and Prolong Diamond Antifade Mountant (Thermo Fischer Scientific) reagent was applied. The slides were covered with a coverslip and dried in the dark at room temperature for 24–48 h. The slides were imaged with a Zeiss LSM 800 confocal microscope (Carl Zeiss) using a 20x objective (NA 0.8) and an Axiocam 506 digital camera. The light source HXP 120 V (Carl Zeiss) and corresponding excitation and emission filters were used: Hoechst 33342 (ex. 335–383 nm; em. 420–470 nm) and PI (ex. 533–558 nm; em. 570–640 nm). The degree of permeabilization at individual voltages was determined from the images using Imaris software (Bitplane) (Fig. 1B).

#### Immunofluorescence labeling and confocal microscopy

After treatment, cells were fixed at different time points (1 h, 4 h or 24 h after treatment). Before fixation, cells were rinsed with Hank’s Balanced Salt Solution supplemented with Ca^2+^ and Mg^2+^ (HBSS^+/+^). Between all staining steps, the cells were washed three times with PBS for 5 min.

For staining of microtubules and actin filaments, a freshly prepared cytoskeleton stabilizing fixation buffer was used (1 × CSB, 2% sucrose, 4% PFA in HBSS^+/+^) to preserve the cytoskeleton structures. Cells were fixed at 37 °C for 10 min. The permeabilization step was performed with permeabilization buffer (PB; 1× PBS, 5% donkey serum, 0.1% or 0.2% Triton X-100 for EA.hy926 or HMEC-1, respectively) for 10 min at room temperature. Cells were then incubated with a primary antibody (anti-α-tubulin, 1 μg/well) in blocking buffer (BB; 2% donkey serum, 22.52 mg/mL glycine) overnight at 4 °C, followed by a 2 h incubation at room temperature with a secondary antibody (1:500) diluted in BB. Actin filaments were stained with Alexa Fluor 546 Phalloidin (Sigma–Aldrich) diluted in BB and incubated at room temperature for 45 minutes. Nuclei were stained with Hoechst 33342 (3 μg/mL; Thermo Fisher Scientific) for 10 min. Slides were then mounted with Prolong Gold Diamond Antifade Mountant (Thermo Fischer Scientific), air-dried for 24 h and sealed with nail polish.

For staining of the cell junction protein VE-cadherin, all fixation and staining steps were performed as described above except for the permeabilization step, which was omitted. Cells were incubated overnight at 4 °C with anti-VE-cadherin (5 μg/well) in BB, followed by incubation with secondary antibody (1:500) for 2 h at room temperature.

Imaging was performed with a Zeiss LSM 800 confocal microscope (Carl Zeiss) and a 63x oil immersion objective (NA 1.4). Lasers providing excitation light at wavelengths of 405, 488, 561 and 640 nm were used for visualization of Hoechst 33342, Alexa Fluor 488, Alexa Fluor 546 and Alexa Fluor 647. The emitted light was collected sequentially with a Gallium Arsenide Phosphide (GaAsP) detector via a variable dichroic at the following wavelengths: 410–617 nm where an additional 620 nm shortpass filter was used, 400–650 nm and 645–700 nm. Different channels were acquired sequentially to prevent the dye bleed through into other channels. The acquired 2D datasets of VE-cadherin and 3D datasets of microtubules and actin filaments were visualized and analyzed with Imaris software (Bitplane). To quantify VE-cadherin, microtubules, and actin filaments, the mean fluorescence intensity of representative images per time point was determined using ImageJ [40, 41]. The background mean intensity was subtracted for each image, and the resulting mean fluorescence intensity was then normalized to the untreated control cells.

#### Intracellular calcium kinetics measurements

A TriStar LB 942 multimode microplate reader (Berthold Technologies) was used to measure and compare ligand- and time-dependent changes in intracellular free Ca^2+^ concentration ([Ca^2+^]_i_) in a population of HMEC-1 and EA.hy926 cells according to previously described protocols [42, 43]. Briefly, trypsinized HMEC-1 or EA.hy926 cells were resuspended in DPBS supplemented with 0.901 mM CaCl_2_, MgCl_2_, glucose and pyruvate to a density of 4 × 10^6^ cells/mL and loaded with 2.5 μM Fura-2-AM for 30 min at ambient temperature (22 ± 2 °C) in the dark. After washing with DPBS, the cells were resuspended in DPBS and seeded in black 96-well plates at a density of 4 × 10^5^ cells/well. The measurement protocol was as follows: The initial measurement period of 40 s (temporal resolution 0.5 s) was used to determine the baseline fluorescence by calculating the ratio of fluorescence emission intensity at 510 nm with excitation at 340 nm and 380 nm (F_340_/F_380_ ratio). Baseline fluorescence obtained in the initial measurement period of 40 s, i.e., before the injection of the test substance, was used to assess basal [Ca^2+^]_i_ in both cell lines. Averaged F_340_/F_380_ ratios obtained before the application of the test substance, reflecting basal [Ca^2+^]_i_, were comparable between both cell lines (Supplementary Fig. S2). Therefore, in our experimental setup, we don’t have experimental evidence of the differences in basal calcium levels between EA.hy926 and HMEC-1. The same approach was also used in a study by Kyoung Sun Park et al. 2016 [44]. Then, individual tested compounds, i.e., ionomycin (10 μM), thapsigargin (1 μM), bradykinin (10 μM), angiotensin II (10 μM), ATP (100 μM) or acetylcholine (300 μM) prepared in DPBS were injected automatically, and the fluorescence intensity was monitored for 300 s. Treatment with DPBS alone was used for mechanostimulation of the cells. Measurements were performed at 30 °C and were completed within 2–3 h after Fura-2-AM loading. In further experiments, cells were treated with bradykinin (10 μM) alone or bradykinin (10 μM) with preincubation with non-selective Ca^2+^ channel inhibitors i.e., 500 μM LaCl_3_ or 500 μM GdCl_3_, which compete with the calcium ions for the negatively charged binding sites and inhibit the transmembrane calcium current [45]. Pretreatment with sarco/endoplasmic reticulum Ca^2+^ ATPase (SERCA) inhibitor thapsigargin (1 μM) was used to deplete of intracellular Ca^2+^ stores. For data analysis, the DPBS-induced signal was considered as background and subtracted from signals obtained with the tested compounds. Changes in the F_340_/F_380_ ratio were used as an index of change in [Ca^2+^]_i_, as described by Grynkiewicz et al. [46].

#### Extraction of total RNA from endothelial cell monolayers and mRNA sequencing

HMEC-1 and EA.hy926 cells were seeded in a T75 flask (VWR) at a density of 3000 cells/cm^2^ and maintained in culture until a confluent monolayer was formed. Then, total RNA was extracted using the Total RNA Kit peqGOLD (VWR) according to the manufacturer’s instructions. Five independent biological replicates were used for each cell line. Qualification of mRNA, library preparation, and sequencing were performed at Novogene Co., Ltd.

Briefly, mRNA was enriched using oligo (dT) beads and randomly fragmented. Then, cDNA was synthesized using random hexamers and reverse transcriptase to synthesize the first strand. Afterwards, a custom second-strand synthesis was performed by addition of dNTPa, RNase H and *Escherichia coli* polymerase I to generate the second strand by nick-translation. The final cDNA library was ready after purification, terminal repair, A-tailing, adaptor ligation, size selection and PCR enrichment. Sequencing of the library was performed with an Illumina NovaSeq 6000 System.

#### Analysis of RNA sequencing data

The obtained raw data were transformed to sequenced reads by base calling and recorded in a FASTQ file containing sequence information (reads) and corresponding sequencing quality information. Then, quality control was performed by determining the error rate, A/T/G/C content distribution and data filtering. Sequences were aligned to the reference genome (human assembly GRCh38/hg38) using HISAT2 and mapped by regions. Read counts, proportional to the actual gene expression levels, were obtained by HTSeq. R software (R Core Team) was used to filter out genes with low expression and normalize the data using the R packages *DESeq2* and *edgeR* (Additional file 1). The Pearson correlation (Supplementary Fig. S3) between samples was calculated, and principal component analysis (PCA) was performed to observe clustering of samples. Differentially expressed genes (DEGs) between EA.hy926 and HMEC-1 samples were assessed by the *Limma* R package, with adjusted *P* value < 0.05 and |log_2_FC| > 1 considered significance thresholds. Additionally, Gene Ontology (GO) analysis and Functional Kyoto Encyclopedia of Genes and Genomes (KEGG) pathway analysis were performed using the *GOseq* and *clusterProfiler* R packages. For comparison of cytoskeleton transcriptome profiles, clusters of human protein–protein interactions (PPIs) related to actin filaments, microtubules and VE-cadherin junctions were obtained from the STRING database (version 12.0) [47]. A needed confidence score greater than 0.4 and size cutoff of no more than 10 interactors was used as the threshold for positive PPIs.

#### Statistical analysis

Statistical analysis of the data was performed with GraphPad Prism 10 software (GraphPad Software). The values were expressed as the arithmetic mean (AM) ± the standard error (SE). Prior to hypothesis testing, normality of the data was assessed for each dataset using Shapiro-Wilk normality test, a method known for its robustness with small sample sizes [48]. In the cytotoxicity assays statistical significance between the control and treatment groups was determined using a t-test or two-way ANOVA with Dunnet’s test. For datasets related to immunofluorescence and Ca^2+^ kinetics, the normality test indicated deviations from normal distribution due to the small sample size. As a result, nonparametric tests were employed for these datasets. Specifically, nonparametric t-tests (Mann-Whitney test) were used for comparisons between two groups, while nonparametric one-way ANOVA (Kruskal-Wallis test) was utilized for comparisons involving multiple groups. Statistical significance was determined based on the following *P*-value thresholds: * *P* < 0.05, ** *P* < 0.01, *** *P* < 0.001, and **** *P* < 0.0001.

## Results

### Optimization of electroporation parameters on formed monolayers of EA.hy926 and HMEC-1 endothelial cells

EA.hy926 and HMEC-1 cells differ in the morphological features of the formed monolayers. Whereas EA.hy926 cells formed an organized and uniform monolayer with evident contact inhibition, the monolayer formed by HMEC-1 cells was denser, tightly packed and overgrown (Fig. 1A). Giemsa staining also revealed a distinct difference between the two monolayers, with EA.hy926 cells exhibiting more intense purple staining than HMEC-1 cells, suggesting that EA.hy926 cells have more abundant cytoplasm. To investigate the effect of CaEP on the two endothelial cell lines, we first determined the optimal voltage of electric pulses for cell permeabilization. The optimal voltage for permeabilization was determined by exposing the cells to electric pulses with increasing voltage by a step of 100 V (Fig. 1B, Supplementary Fig. S4), while the cell survival was determined by exposing the cells to electric fields of 800, 900, 1000 and 1300 V/cm (Fig. 1C). Both the permeabilization and cell survival of ECs after exposure to increasing voltage showed that EA.hy926 cells are more sensitive to treatment than HMEC-1 cells. Based on the obtained results, a voltage of 645 V (1000 V/cm) was chosen for further experiments, as this treatment led to equivalent permeabilization of EA.hy926 and HMEC-1 cells, with minimal effects on cell survival and morphology (Fig. 1B, Supplementary Fig. S4).

### Cytotoxicity of CaEP in HMEC-1 and EA.hy926 cells

**Fig. 2:**
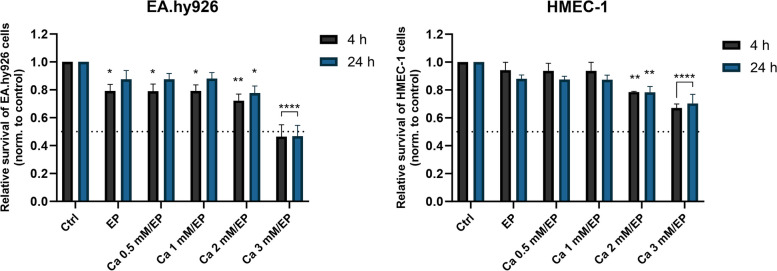
Cytotoxicity of CaEP in EA.hy926 and HMEC-1 cells. Graphs show the relative survival of EA.hy926 and HMEC-1 cells 4 and 24 h after CaEP. Data were normalized to control untreated cells and presented as AM ± SEM (*n* ≥ 4). The dotted horizontal line marks the relative survival of 0.5. Statistical significance was determined by two-way ANOVA and compared to the control (Ctrl). A *P* value of < 0.05 was considered to be statistically significant (*P < 0.05, **P < 0.01, ****P < 0.0001 vs control (Ctrl))

After determining the optimal voltage of electric pulses for EC permeabilization, we evaluated how increasing [Ca^2+^]_i_ affects the survival of EA.hy926 and HMEC-1 cells grown in monolayers. We performed a viability assay at different time points after treatment with CaEP in the presence of different extracellular Ca^2+^ concentrations, i.e., 0 mM (control (Ctrl)), 0.5 mM, 1 mM, 2 mM and 3 mM CaCl_2_ (Fig. 2). EP was used to permeabilize cell membranes and induce influx of Ca^2+^ ions in a concentration gradient-dependent manner. Viability assessment of control or treated cells was performed at 4 h or 24 h after treatment. In the case of 0.5 mM or 1 mM CaEP, no significant effect on the survival of either EA.hy926 or HMEC-1 cells was observed. After CaEP in the presence of 2 mM Ca^2+^, both cell lines exhibited an approximately 20% drop in viability. This effect was further potentiated at a Ca^2+^ concentration of 3 mM, resulting in a viability drop in EA.hy926 cells below 50%. A significant difference between EA.hy926 and HMEC-1 cells was observed at a Ca^2+^ concentration of 3 mM, suggesting that HMEC-1 cells can eliminate cytosolic Ca^2+^ more efficiently.

### Effects of CaEP with increased extracellular calcium concentration on cytoskeletal organization in EA.hy926 and HMEC-1 cells

**Fig. 3:**
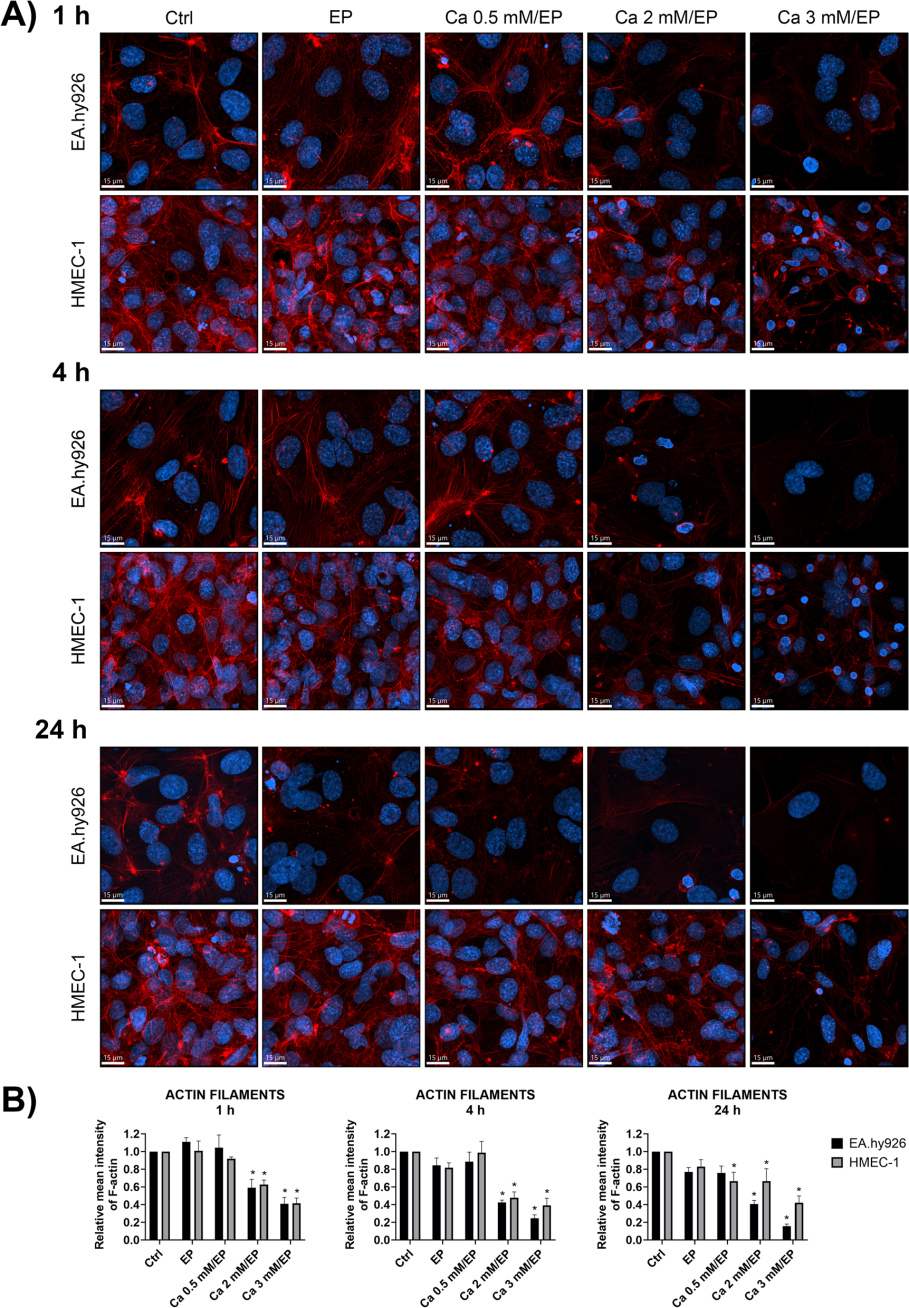
Effect of increased intracellular Ca^2+^ concentration by CaEP on actin filaments in EA.hy926 and HMEC-1 cells. **A** Representative confocal images show changes in the F-actin cytoskeleton of both EA.hy926 and HMEC-1 cells at 1, 4 and 24 h after EP alone or after CaEP in the presence of 0.5, 2 or 3 mM Ca^2+^. **B** The plots represent quantification of changes in the average F-actin mean fluorescence intensity after CaEP at 1, 4 and 24 h time points. Data were normalized to the control and presented as AM ± SEM (*n* ≥ 3). Statistical significance was determined by a nonparametric one-way ANOVA (Kruskal-Wallis). A *P* value of < 0.05 was considered to be statistically significant (*P < 0.05, ** *P* < 0.01, *** *P* < 0.001, and **** *P* < 0.0001 vs control (Ctrl)). Scale bar: 15 μm

**Fig. 4:**
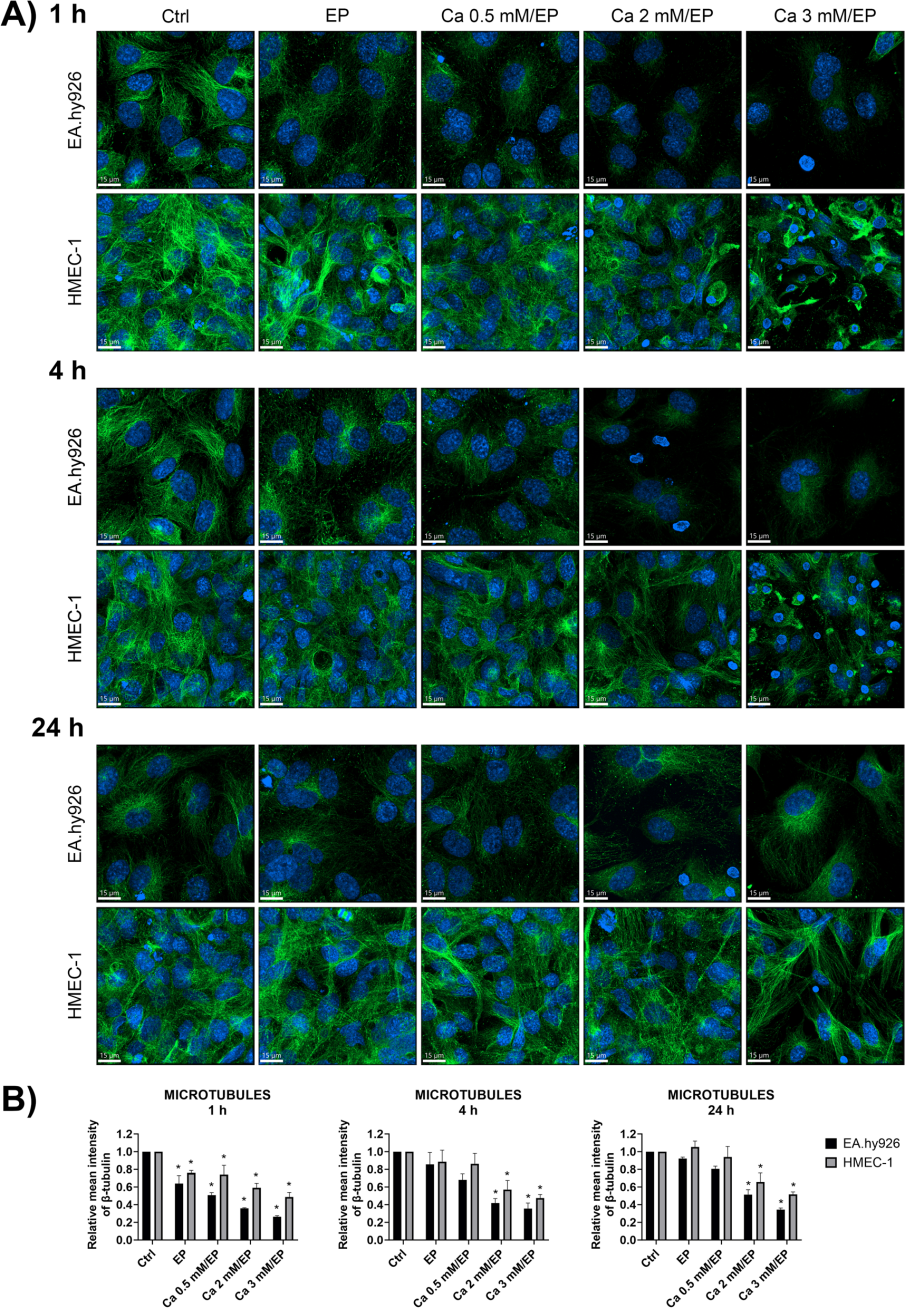
Effects of increased intracellular Ca^2+^ concentration by CaEP on microtubules in EA.hy926 and HMEC-1 cells. **A** Representative confocal images show changes in the cytoskeleton α-tubulin in EA.hy926 and HMEC-1 cells at 1, 4 and 24 h after EP alone or after CaEP in the presence of 0.5, 2 or 3 mM Ca^2+^. **B** The plots represent quantification of the changes in average α-tubulin mean fluorescence intensity after CaEP at 1, 4 and 24 h time points. Data were normalized to the control and presented as AM ± SEM (*n* ≥ 3). Statistical significance was determined using a nonparametric one-way ANOVA (Kruskal-Wallis). A *P* value of < 0.05 was considered to be statistically significant (*P < 0.05, and ** *P* < 0.01 vs control (Ctrl)). Scale bar: 15 μm

**Fig. 5:**
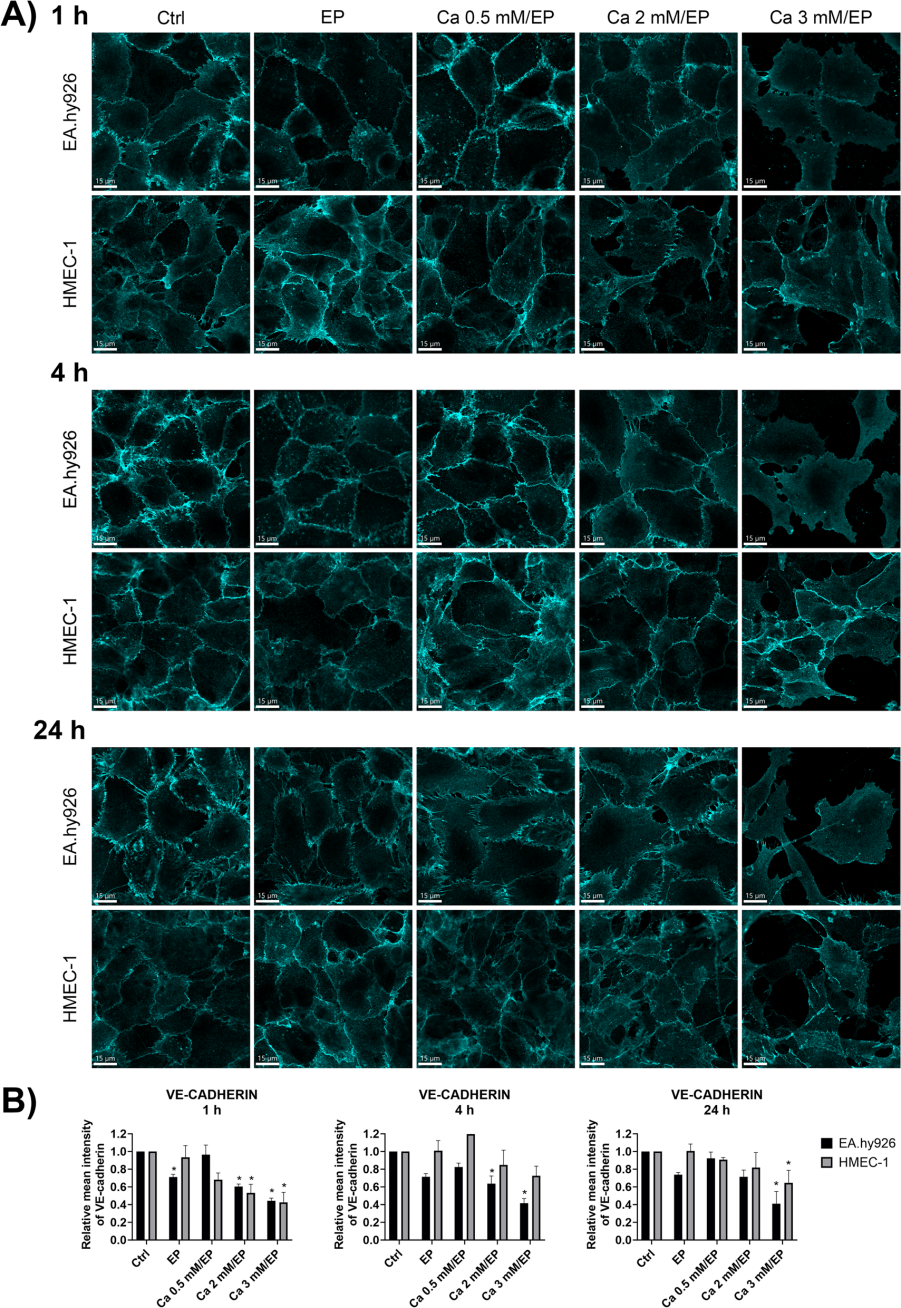
Effects of increased intracellular Ca^2+^ concentration by CaEP on cell–cell junctions in EA.hy926 and HMEC-1 cells. **A** Representative confocal images show changes in the cell–cell adhesion protein VE-cadherin in EA.hy926 and HMEC-1 cells at 1, 4 and 24 h after EP alone or after CaEP in the presence of 0.5, 2 or 3 mM Ca^2+^. **B** The plots represent quantification of the changes in average VE-cadherin mean fluorescence intensity after CaEP at 1, 4 and 24 h time points. Data were normalized to the control and presented as AM ± SEM (*n* ≥ 3). Statistical significance was determined using a nonparametric one-way ANOVA (Kruskal-Wallis). A *P* value of < 0.05 was considered to be statistically significant (*P < 0.05, ** *P* < 0.01, and *** *P* < 0.001 vs control (Ctrl)). Scale bar: 15 μm

We next determined the effect of increasing the intracellular Ca^2+^ concentration on the cytoskeleton in both endothelial cell lines, EA.hy926 and HMEC-1, grown in monolayers. Staining with fluorescently labeled phalloidin, α-tubulin and VE-cadherin at different time points after treatment with CaEP in the presence of different extracellular Ca^2+^ concentrations, i.e., 0 mM (control (Ctrl)), 0.5 mM, 2 mM and 3 mM CaCl_2_ (Figs. 3, 4 and 5).

There was a visible difference between EA.hy926 and HMEC-1 cells in the actin cytoskeleton (Fig. 3). In untreated confluent EA.hy926 cells, actin structures were organized as thick cortical rings with a few thin actin bundles extending through the cell body. In contrast to EA.hy926 cells, HMEC-1 cell actin filaments extended throughout the cell body, with no distinct clustering at the cell margin (Fig. 3A). Electroporation (EP) without the presence of Ca^2+^ ions was used as a control to determine the effects of the electric field on the cytoskeleton, where we observed that at given time points, EP alone did not significantly affect the actin cytoskeleton. At 0.5 mM Ca^2+^, no effect on actin filaments was observed. The mean fluorescence intensity of actin filaments was significantly reduced in the presence of 2 mM and 3 mM Ca^2+^ (Fig. 3B). EA.hy926 cells were more susceptible to major increases in intracellular Ca^2+^, as a higher rate of cell death was observed at 3 mM Ca^2+^ compared to HMEC-1 cells (Fig. 2A). This was also indicated by the cell detachment shown in the representative confocal images, in which the surviving cells flattened and formed multidirectional stress fibers (Fig. 3A). As for EA.hy926 cells detachment and flattening of the surviving cells was also observed for HMEC-1 cells but to a lesser extent. EP-treated HMEC-1 cells showed cell swelling at 1 h and 4 h after treatment as well as a decline in the mean fluorescence intensity of the actin cytoskeleton; however, at 24 h posttreatment, EP-treated HMEC-1 cells recovered.

Both endothelial cell lines in the control groups displayed intact microtubule networks (Fig. 4). In EA.hy926 cells, microtubules were more concentrated around the nucleus, indicating the formation of a microtubule-organizing center (centrosome), whereas actin filaments formed a network predominantly in the periphery (cf. Figs. 3A and 4A). The mean fluorescence intensity of stained microtubules was reduced 1 h after EP alone, with microtubules concentrating around nuclei in fused cells and some fibers extending to the periphery of the cell (Fig. 4B). After EP and in the presence of Ca^2+^, microtubules in EA.hy926 cells became fragmented and extended throughout the cell, indicating disruption of these cytoskeletal structures (Fig. 4A). At 4 h time point, microtubules returned to their normal arrangement, primarily concentrated around the nucleus. This was consistent with an increased mean fluorescence intensity of microtubules in EA.hy926 cells in some treatment groups compared to 1 h after treatment. However, a significant decrease in mean fluorescence intensity was observed in EA.hy926 cells 4 h after treatment with 2 and 3 mM CaEP. At 24 h after treatment, the mean fluorescence intensity of microtubules in EA.hy926 cells after 3 mM CaEP was still significantly reduced compared to the control.

In HMEC-1 cells, the centrosome was not clearly detectable, with microtubules extending throughout the cell. Some fragmentation and disruption of microtubules was visible 1 h after treatment with EP alone; however, after 4 h, microtubules reorganized back to their original state (Fig. 4A). A similar increase in mean fluorescence intensity at 4 h time point as for EA.hy926 cells was observed for HMEC-1 cells, but only after treatment with EP and 0.5 mM CaEP. When Ca^2+^ was present in the EP buffer, this was not observed, indicating that higher Ca^2+^ concentrations may hinder the process of microtubule reformation. At 3 mM Ca^2+^, detachment and cell death of HMEC-1 cells were observed, but to a lesser extent compared to EA.hy926 cells.

The effects of EP on cell–cell junctions have already been described [49–52]. To further confirm the effects of elevated intracellular Ca^2+^ induced by CaEP on cell–cell junctions, immunofluorescence staining of EA.hy926 and HMEC-1 monolayers with an anti-VE-cadherin antibody was performed (Fig. 5). Monolayers were fixed and stained at different time points, i.e., 1 h, 4 h and 24 h after treatment. As for actin filaments and microtubules, the cell–cell junctions of EA.hy926 cells were also more affected by CaEP than the cell–cell junctions of HMEC-1 cells (Fig. 5A). At 1 h time point, both cell lines showed a significant decrease in the mean fluorescence intensity of VE-cadherin, but only after 3 mM CaEP (Fig. 5B). While HMEC-1 cells were not significantly affected 4 h after treatment, EA.hy926 cells showed a significantly reduced mean fluorescence intensity of VE-cadherin after EP, 2 and 3 mM CaEP (Fig. 5B). At 4 h and 24 h after treatment, the junctions in HMEC-1 cells became more chaotic in shape, as indicated by VE-cadherin staining (Fig. 5A). Compared to HMEC-1 cells, the mean fluorescence intensity of VE-cadherin junctions in EA.hy926 cells was still significantly reduced compared to the control (Fig. 5B). For more precise quantification of the VE-cadherin signal at cell-cell junctions in EA.hy926 and HMEC-1 cells, the line scan profile of maximum fluorescence intensity was determined at all time points (Supplementary Fig. S5). While VE-cadherin in HMEC-1 cells appeared unaffected, EA.hy926 cells exhibited sensitivity. Specifically, at 1 h time point, only EA.hy926 demonstrated a significant decrease in maximum intensity of VE-cadherin following exposure to 3 mM CaEP. At the 4 and 24 h time points, EA.hy926 cells displayed a significantly reduced maximum intensity of VE-cadherin after EP and 3 mM CaEP. Importantly, the average ratios (PM/CS) of the fluorescence signals of VE-cadherin on the plasma membrane (PM) to the VE-cadherin fluorescence signals of the cytosol (CS) did not significantly change at any time point or treatment.

### Intracellular Ca^2+^ activity in EA.hy926 and HMEC-1 cells

**Fig. 6:**
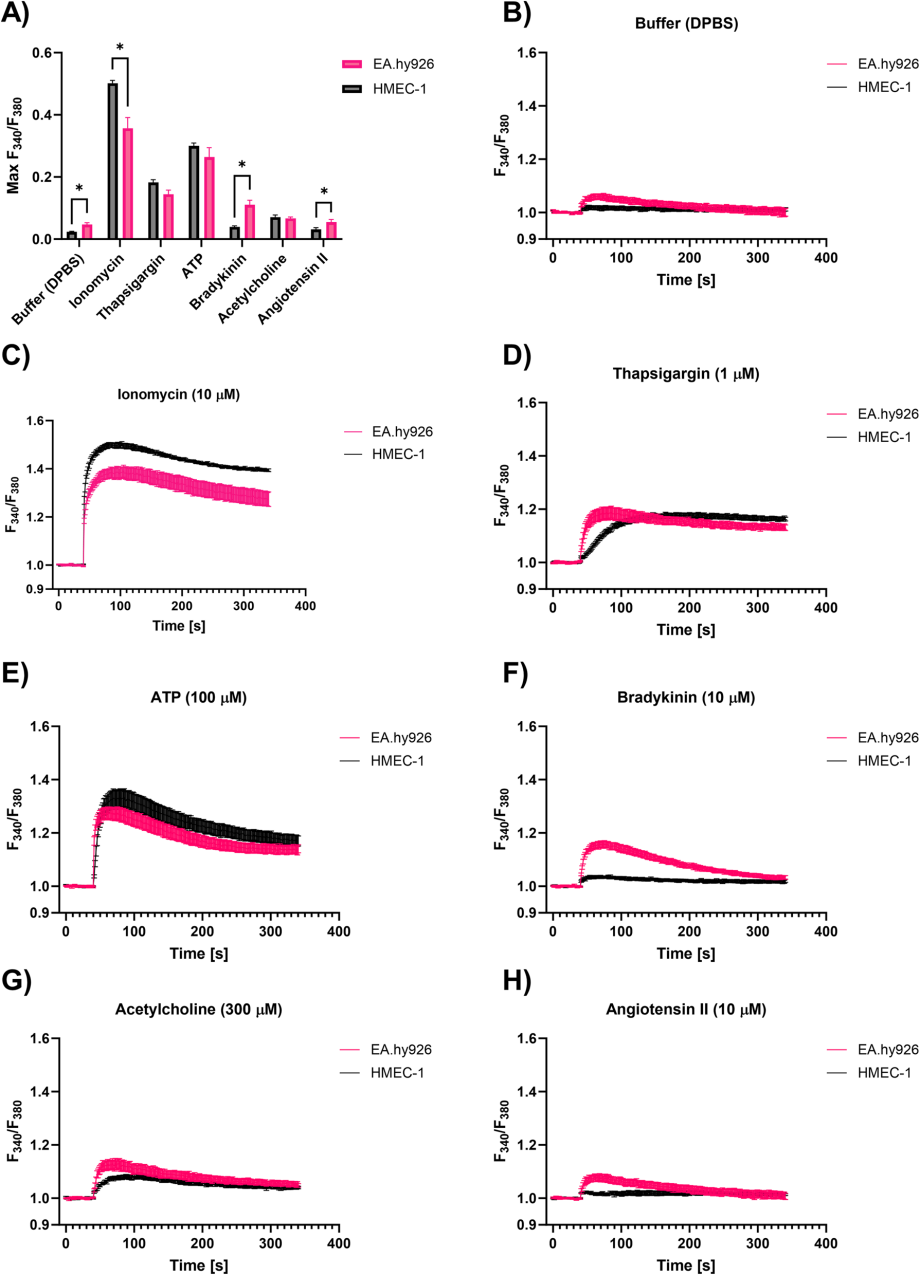
Fluorometric kinetic measurements of [Ca^2+^]_i_ in EA.hy926 and HMEC-1 cells. **A** Summary bar graph showing the average maximum increase in the F_340_/F_380_ ratio in HMEC-1 and EA.hy926 cells labeled with Fura-2-AM and exposed to flow of buffer (DPBS), ionomycin, thapsigargin, ATP, bradykinin, acetylcholine and angiotensin II. Changes in the F_340_/F_380_ ratio induced by DPBS were subtracted in all subsequent measurements to obtain ligand-specific responses. Bars display AM of maximum amplitude in F_340_/F_380_ ratio ± SE (*n* ≥ 8). Statistical significance was determined using a nonparametric t-test (Mann-Whitney). A *P* value of < 0.05 was considered to be statistically significant (*P < 0.05 between EA.hy926 and HMEC-1 cells). Time course of the changes in the F_340_/F_380_ ratio during the injection of **B**) buffer (DPBS), **C**) ionomycin (10 μM), **D**) thapsigargin (1 μM), E) ATP (100 μM), **F**) bradykinin (10 μM), **G**) acetylcholine (300 μM) and **H**) angiotensin II (10 μM) in EA.hy926 and HMEC-1 cells loaded with the Ca^2+^-sensitive fluorescent dye Fura-2-AM. Data are presented as AM ± SEM (*n* ≥ 9)

Fluorometric kinetic measurements of intracellular free Ca^2+^ concentration ([Ca^2+^]_i_) were performed on EA.hy926 and HMEC-1 cells under various experimental conditions. We compared changes in [Ca^2+^]_i_ in response to mechanical stress, Ca^2+^ influx from the extracellular space and ligand-mediated mobilization of intracellular stores (Fig. 6).

To determine the Ca^2+^ kinetics regulated by mechanosensing ion channels, EA.hy926 and HMEC-1 cells were exposed to mechanical stress induced by injection of Dulbecco’s phosphate-buffered saline (DPBS). Although a minimal response was observed for both cell lines, we found that EA.hy926 cells had a significantly higher response to mechanical stress than HMEC-1 cells (Fig. 6A and B). This suggests that EA.hy926 cells have higher expression of mechanosensing ion channels (such as TRPVs, Piezo1 and PKD1/2) than HMEC-1 cells. In all subsequent measurements, changes induced by DPBS were subtracted to obtain ligand-specific responses.

The ionophore ionomycin (10 μM) served as a positive control, as it induces a fast transient increase in intracellular Ca^2+^ by facilitating the transport of Ca^2+^ across the plasma membrane and through releasing Ca^2+^ from its intracellular stores. The kinetics of ionomycin-induced [Ca^2+^]_i_ were comparable between the cell lines; however, the maximum amplitude response in HMEC-1 cells was significantly higher (Fig. 6A and C). The depletion of intracellular Ca^2+^ stores via the inhibition of SERCA pumps using thapsigargin (1 μM) exhibited distinct kinetics, with a faster rate observed in EA.hy926 cells. However, the amplitude of the response was comparable between the two cell lines (Fig. 6A and D).

Next, the involvement of vasoactive receptor agonists, i.e., ATP (100 μM), bradykinin (10 μM), acetylcholine (300 μM) and angiotensin II (10 μM), in [Ca^2+^]_i_ regulation was tested (Fig. 6A and E-H). These agonists can stimulate ionotropic P2X receptors (ATP) or G-protein coupled receptors (GPCR)-mediated responses (bradykinin, angiotensin II, acetylcholine and ATP), including the production of inositol 1,4,5-trisphosphate (IP_3_), which leads to intracellular release of Ca^2+^ from the endoplasmic reticulum (ER) through IP_3_ receptor (IP_3_R) Ca^2+^ channels. ATP induced the highest increase in [Ca^2+^]_i_, and this response exhibited comparable kinetics and maximum amplitude in both cell lines (Fig. 6A and E). Changes in [Ca^2+^]_i_ induced by bradykinin, acetylcholine and angiotensin II were substantially lower than those induced by ATP (Fig. 6A, F-H). Among them, bradykinin induced the highest maximum [Ca^2+^]_i_ amplitude response. Both bradykinin and angiotensin II induced significantly higher [Ca^2+^]_i_ in EA.hy926 cells than in HMEC-1 cells (Fig. 6A, F and H), thus suggesting very low/negligible expression of bradykinin and angiotensin receptors in HMEC-1 cells. The same trend was observed for an otherwise very low response to acetylcholine (Fig. 6A and G).

**Fig. 7:**
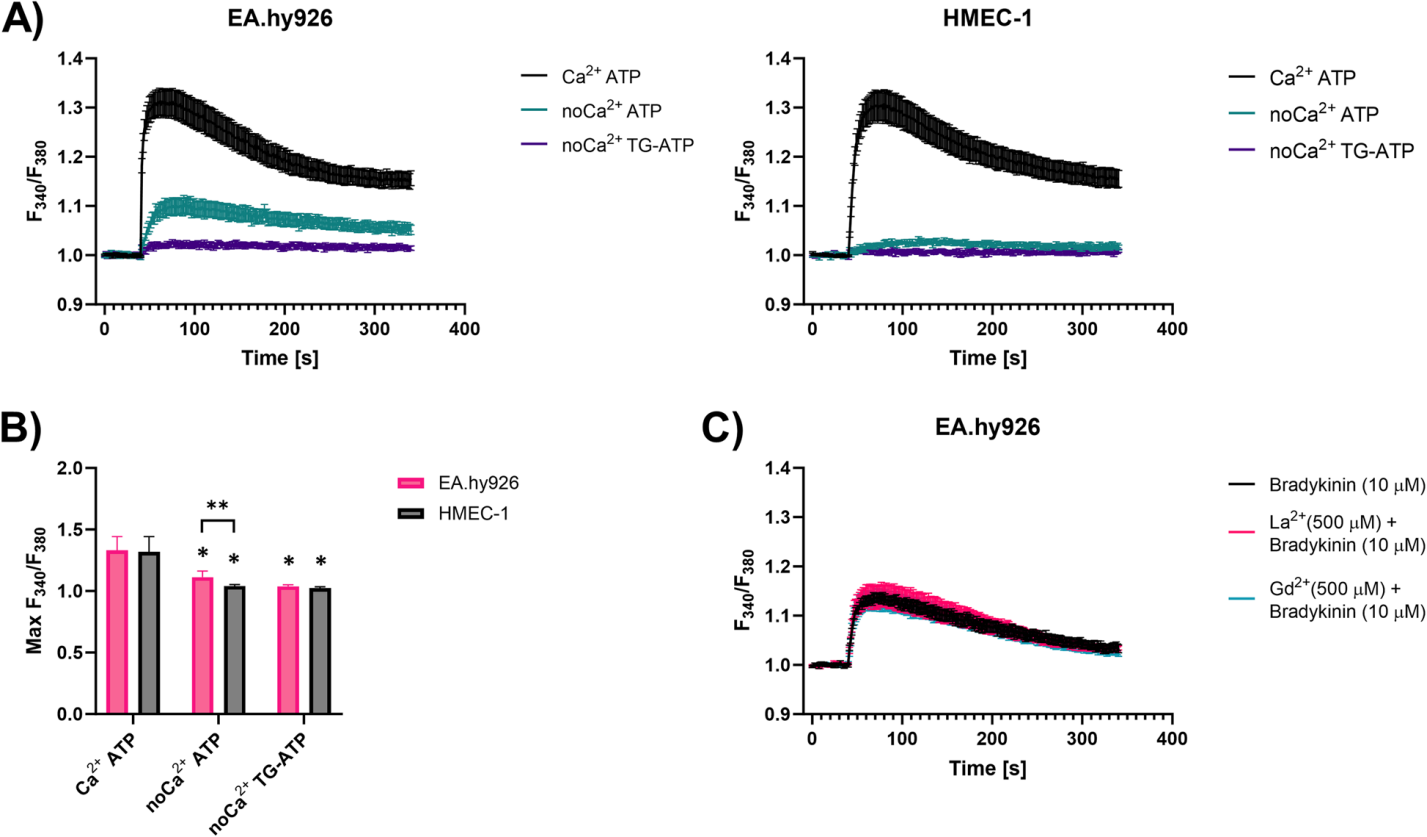
Fluorometric kinetic measurements of [Ca^2+^]_i_ in EA.hy926 and HMEC-1 cells. A) Time course of the changes in the F_340_/F_380_ ratio in EA.hy926 and HMEC-1 cells during the injection of ATP (100 μM) with or without preincubation with thapsigargin (TG) (1 μM). These changes were followed in the presence or absence of Ca^2+^ (no Ca^2+^). Data are presented as AM ± SEM (*n* ≥ 9). B) Summary bar graph showing the average maximum increase in the F_340_/F_380_ ratio in HMEC-1 and EA.hy926 cells treated as described in A). Bars display AM of maximum amplitude in F_340_/F_380_ ratio ± SE (n ≥ 9). Statistical significance was determined using a nonparametric t-test (Mann-Whitney). A *P* value of < 0.05 was considered to be statistically significant (*P < 0.05 vs Ca^2+^ ATP, **P < 0.05 between EA.hy926 and HMEC-1 cells). C) Time course of the changes in the F_340_/F_380_ ratio in EA.hy926 and HMEC-1 cells during the injection of bradykinin (10 μM) alone or bradykinin (10 μM) with preincubation with LaCl_3_ (500 μM) or GdCl_3_ (500 μM)

To determine the contribution of intracellular Ca^2+^ stores versus external Ca^2+^ influx to the increase in cytosolic Ca^2+^ in EA.hy926 and HMEC-1 cells, we monitored changes in [Ca^2+^]_i_ in response to ATP (100 μM) with or without preincubation with thapsigargin (1 μM) (Fig. 7A). When ATP was applied in the presence of Ca^2+^ in the external medium, both cell lines exhibited an expected increase in [Ca^2+^]_i_, whereas in Ca^2+^-free medium, the ATP-mediated increase in [Ca^2+^]_i_ was significantly lower in EA.hy926 cells and almost absent in HMEC-1 cells (Fig. 7B). In Ca^2+^-free medium, neither cell line exhibited a response to ATP when pretreated with thapsigargin. Taken together, it appears that in EA.hy926 cells, the increase in [Ca^2+^]_i_ in response to ATP is induced by i) influx through ionotropic P2X receptors and ii) release from the intracellular ER store, which is triggered by the activation of metabotropic G_αq/11_-coupled P2Y receptors. In contrast, the increase in [Ca^2+^]_i_ in HMEC-1 cells in response to ATP seems to be driven only via the activation of ionotropic P2X receptors. Finally, the effect of LaCl_3_ (500 μM) and GdCl_3_ (500 μM), which inhibit Ca^2+^ influx by blocking various cation-selective channels (i.e. voltage-gated calcium, TRP and stretch-activated channels), was tested (Fig. 7C). The presence of neither of these blockers interfered with bradykinin-mediated changes in [Ca^2+^]_i_ in both EA.hy926 and HMEC-1 cells, thus providing evidence for G_αq/11_-coupled bradykinin receptor-mediated increases in [Ca^2+^]_i_.

### Global transcriptomic profiling of the human endothelial cell lines EA.hy926 and HMEC-1

**Fig. 8:**
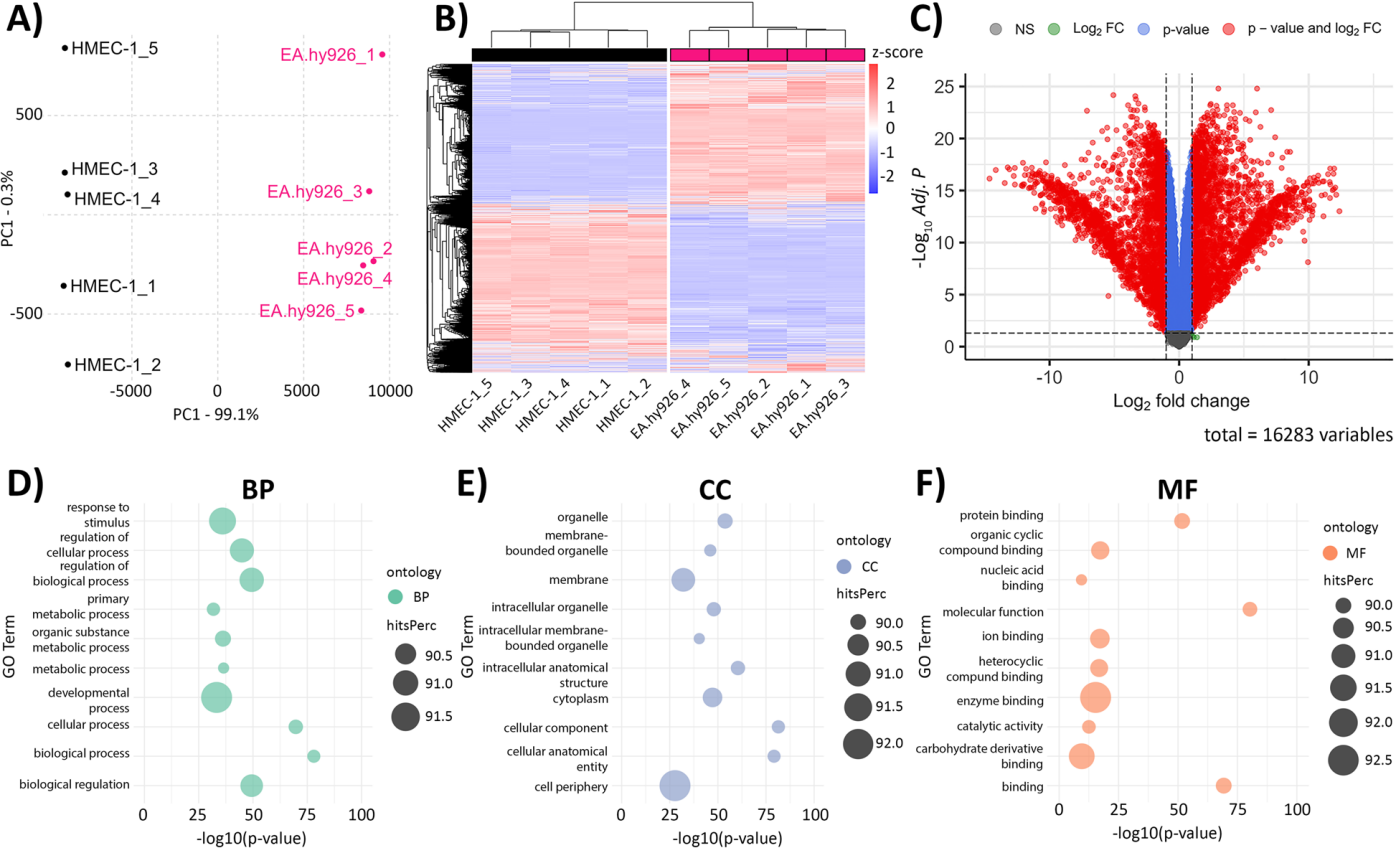
Comparison of global transcriptomic profiles of EA.hy926 and HMEC-1 cells. **A** PCA plot showing clustering of EA.hy926 and HMEC-1 samples based on normalized gene expression levels. PC1 - principal component 1, PC2 - principal component 2. **B** Heatmap presenting unsupervised hierarchical clustering of all genes across EA.hy926 and HMEC-1 samples. Normalized expression levels were scaled to Z-scores for each gene. **C** Volcano plot showing DEGs in EA.hy926 samples compared to HMEC-1 samples. Genes with adjusted *P* values of < 0.05 and |log_2_FC| > 1 were considered to be statistically significant. A total of 16,283 genes were analyzed. NS - not significant, FC - fold change. **D**-**F** Dot plots presenting the top 10 significantly enriched GO processes based on hitsPerc (n (DEGs in category)/n (all genes in category) * 100). A P value < 0.05 was considered significant. BP - biological processes (**D**), CC - cellular components (**E**), MF - molecular functions (**F**). Five replicates per group in all analyses are presented

To understand the underlying differences in response to CaEP and agonists of Ca^2+^ channels between the human endothelial cell lines EA.hy926 and HMEC-1, RNA-seq profiles of untreated EA.hy926 and HMEC-1 cells were compared. Principal component analysis (PCA) showed clustering of EA.hy926 cells apart from HMEC-1 cells, with principal component 1 (PC1 = 99.1%) driving the majority of the variance (Fig. 8A). To visualize the differences in gene expression at the whole transcriptome level, we generated a heatmap showing the expression of normalized gene counts across the samples (Fig. 8B, Additional file 1). Differential gene expression (DGE) analysis showed 5366 significantly differentially expressed genes (adjusted *P* value < 0.05, |log_2_FC| > 1), of which 2407 genes were upregulated in EA.hy926 cells compared to HMEC-1 cells. In contrast, 2959 genes were downregulated in EA.hy926 cells (Fig. 8C).

DEGs between the EA-hy926 and HMEC-1 cell lines were then categorized into three enriched categories based on gene ontology (GO) analysis: Biological Process (BP), Cellular Component (CC) and Molecular Function (MF) (Fig. 8D-F, Additional files 2, 3 and 4). In the BP GO category, we found 25 significantly (*P* value < 0.05) enriched processes, including processes related to the regulation of cellular and molecular processes, metabolism, and development (Fig. 8D, Additional file 2). In the CC GO category, 233 processes were significantly enriched (P value < 0.05), most of which were related to membrane and intracellular organelles (Fig. 8E, Additional file 3). Among 196 significantly enriched processes (P value < 0.05) in the MF GO category, processes involved in protein binding were found (Fig. 8F, Additional file 4). To assess the enrichment of signaling pathways in EA.hy926 cells compared to HMEC-1 cells, Functional Kyoto Encyclopedia of Genes and Genomes (KEGG) pathway analysis was performed, which showed 149 significantly (adjusted *P* value < 0.05) overrepresented signaling pathways. The top 20 significant pathways are shown in (Supplementary Fig. S6).

### Cytoskeleton-related transcriptome profiles of EA.hy926 and HMEC-1 cells

**Fig. 9:**
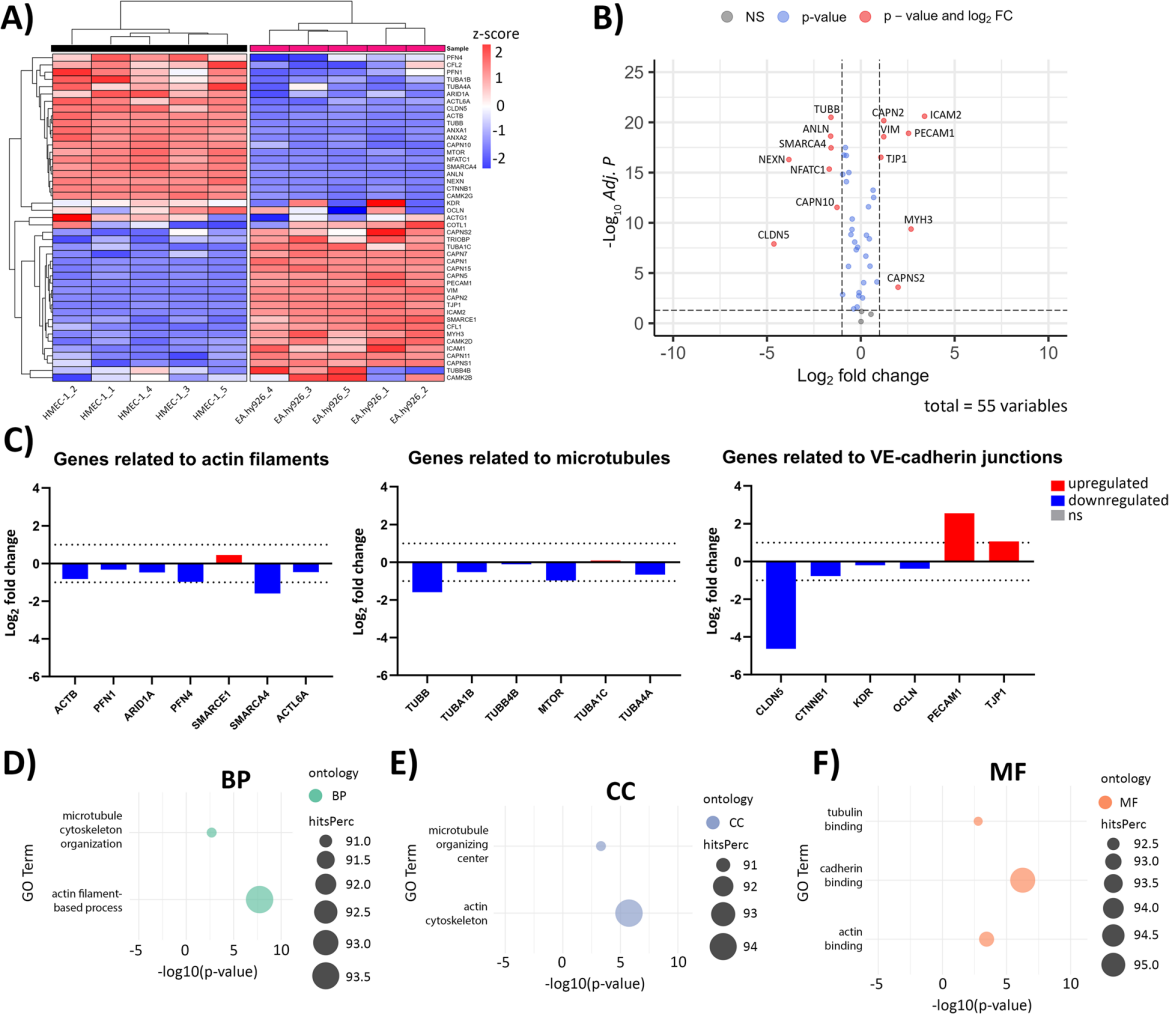
Comparison of cytoskeleton-related RNA profiles of EA.hy926 and HMEC-1 cells. **A** Heatmap showing unsupervised hierarchical clustering of cytoskeleton-related genes across EA.hy926 and HMEC-1 samples. Normalized expression levels were scaled to Z-scores for each gene. **B** Volcano plot showing DEGs related to the cytoskeleton in EA.hy926 samples compared to HMEC-1 samples. Genes with adjusted *P* values of < 0.05 and |log_2_FC| > 1 were considered to be statistically significant. A total of 55 genes were analyzed. NS - not significant, FC - fold change. **C** Bar plots showing log_2_FC of DEGs involved in potential PPIs related to actin filaments, microtubules and VE-cadherin junctions according to the STRING database. A *P* value of < 0.05 and |log_2_FC| > 0 were considered to be statistically significant (red – upregulated genes, blue – downregulated genes, ns – not significant). Dotted horizontal lines mark log_2_FC > 1 and log_2_FC < − 1. D-F) Dot plots presenting significantly enriched GO processes related to the cytoskeleton and based on hitsPerc (n (DEGs in category)/n (all genes in category) * 100). A *P* value < 0.05 was considered significant. BP - biological processes (**D**), CC - cellular components (**E**), MF - molecular functions (**F**). Five replicates per group in all analyses are presented

To explain the differences in the expression of F-actin, β-tubulin, and VE-cadherin after CaEP observed by immunofluorescence staining, cytoskeleton-related transcriptomic profiles of the EA.hy926 and HMEC-1 cell lines were compared (Fig. 9A). Out of 55 analyzed cytoskeleton-related genes, *ICAM2* (log_2_FC = 3.40)*, MYH3* (log_2_FC = 2.68) and *PECAM1* (log_2_FC = 2.54) were the top three significantly upregulated (adjusted P value < 0.05, log_2_FC > 1) in EA.hy926 cells compared to HMEC-1 cells (Fig. 9B). In contrast, *CLDN5* (log_2_FC = − 4.63), *NEXN* (log_2_FC = − 3.83) and *NFATC-1* (log_2_FC = − 1.68) were found to be the top three significantly downregulated genes (adjusted P value < 0.05, log_2_FC < − 1) in EA.hy926 cells (Fig. 9B). Among genes related to the regulation of cytoskeletal integrity, VIM (log_2_FC = 1.22), CAPN2 (log_2_FC = 1.22), and CAPNS2 (log_2_FC = 1.99) were significantly upregulated, while CAPN10 (log_2_FC = − 1.27) was significantly downregulated. To identify additional gene interactions that could be altered in the regulation of the cytoskeleton, clusters of human protein–protein interactions (PPIs) related to actin filaments, microtubules and VE-cadherin junctions were obtained from the STRING database [47]. DEGs involved in potential PPIs are presented as individual bar plots (Fig. 9C), where the majority of genes related to actin filaments, microtubules and VE-cadherin junctions were found to be significantly downregulated in EA.hy926 cells compared to HMEC-1 cells. Among these *SMARCA4* (log_2_FC = − 1.59)*, TUBB* (log_2_FC = − 1.59) and *CLDN5* were the most downregulated. *PECAM1* and TJP1 (log_2_FC = 1.07) were the two most upregulated genes related to VE-cadherin junctions.

GO analysis showed significantly enriched GO processes related to the cytoskeleton, including Actin filament-based process (BP), Microtubule cytoskeleton organization (BP), Actin cytoskeleton (CC), Microtubule organizing center (CC), Actin binding (MF), Tubulin binding (MF), and Cadherin binding (MF) (Fig. 9D-F). Additionally, KEGG analysis revealed significant enrichment of Regulation of actin cytoskeleton (Supplementary Fig. S7).

### Ca^2+^ signaling-related transcriptome profiles of EA.hy926 and HMEC-1 cells

**Fig. 10:**
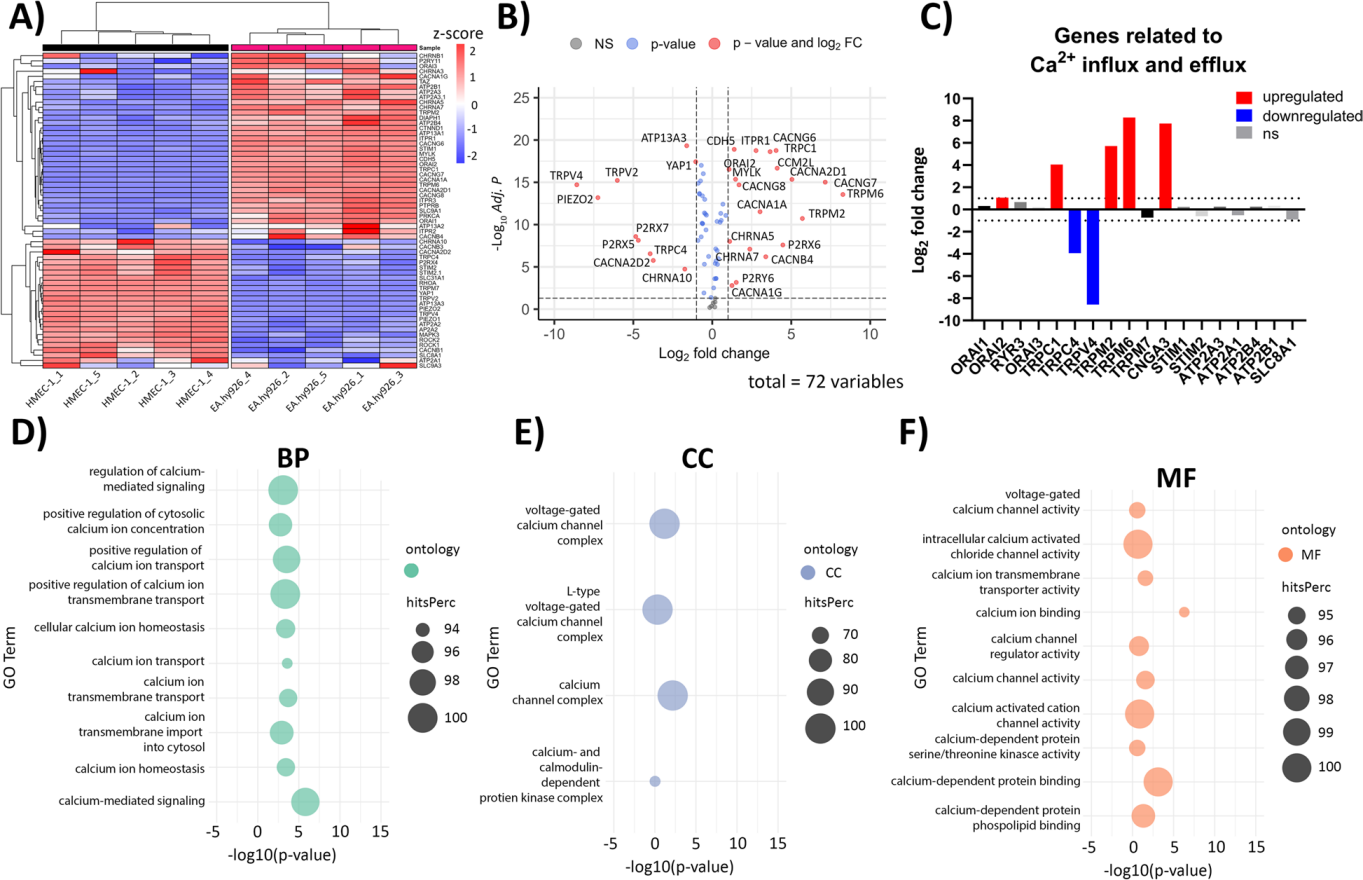
Comparison of the Ca^2+^ signaling-related transcriptomes of EA.hy926 and HMEC-1 cells. **A** Heatmap showing unsupervised hierarchical clustering of Ca^2+^ signaling-related genes across EA.hy926 and HMEC-1 samples. Normalized expression levels were scaled to Z-scores for each gene. **B** Volcano plot presenting DEGs related to Ca^2+^ signaling in EA.hy926 samples compared to HMEC-1 samples. Genes with adjusted *P* values of < 0.05 and |log_2_FC| > 1 were considered to be statistically significant. A total of 72 genes were tested. NS - not significant, FC - fold change. **C** Bar plot showing the expression of genes related to channels involved in Ca^2+^ influx and efflux. A P value of < 0.05 and |log_2_FC| > 0 were considered to be statistically significant (red – upregulated genes, blue – downregulated genes, ns – not significant). Dotted horizontal lines mark log_2_FC > 1 and log_2_FC < − 1. **D**-**F** Dot plots presenting the top 10 significantly enriched GO processes and four significantly enriched GO processes related to Ca^2+^ signaling based on hitsPerc (n (DEGs in category)/n (all genes in category) * 100). A P value < 0.05 was considered significant. BP - biological processes (**D**), CC - cellular components (**E**), MF - molecular functions (**F**). Five replicates per group in all analyses are presented

Differences in [Ca^2+^]_i_ between EA.hy926 and HMEC-1 cells after exposure to agonists of Ca^2+^ channels were investigated by focusing on specific genes and pathways related to Ca^2+^ signaling and transport through different cellular compartments. Heatmap visualization of Ca^2+^ signaling-related genes showed that EA.hy926 samples indeed differed from HMEC-1 samples in the expression of specific gene subsets (Fig. 10A). Among 72 analyzed Ca^2+^signaling-related genes, 19 were significantly upregulated (adjusted *P* value < 0.05, log_2_FC > 1), with *TRPM6* (log_2_FC = 8.27), *CACNG7 (*log_2_FC = 7.15), and *TRPM2* (log_2_FC = 5.71) being the top three upregulated genes in the EA.hy926 cell line compared to the HMEC-1 cell line (Fig. 10B). On the other hand, *TRPV4* (log_2_FC = − 8.58), *PIEZO2* (log_2_FC = − 7.24), and *TRPV2* (log_2_FC = − 6.01) were the top three (out of 10) significantly downregulated (adjusted P value < 0.05, log_2_FC < − 1) Ca^2+^signaling-related genes (Fig. 10B). We then analyzed genes related to Ca^2+^ channels, which are associated with Ca^2+^ signaling in ECs [11, 53]. We divided these genes into genes that form Ca^2+^ channels involved in either Ca^2+^ entry into the cytoplasm or Ca^2+^ efflux from the cytoplasm into the ER or extracellular space. Among genes involved in Ca^2+^ influx, the EA.hy926 cell line showed significantly higher expression (adjusted P value < 0.05, log_2_FC > 1) of the *ORAI2*, *TRPC1*, *TRPM2*, CNGA3 and *TRPM6* genes and significantly lower expression (adjusted *P* value < 0.05, log_2_FC < − 1) of *TRPV4* and *TRPC4* than the HMEC-1 cell line (Fig. 10C). In contrast, there was no significant difference in genes (*ATP2A3*, *ATP2A1*, *ATP2B4*, *ATP2B1*, *SLC8A1*) involved in Ca^2+^ efflux between the cell lines (Fig. 10C). Additionally, RNA-seq showed significantly different expression (adjusted P value < 0.05, |log_2_FC| > 1) of receptor genes involved in the regulation of Ca^2+^ signaling [54], including those for members of GPCR family i.e., metabotropic glutamate receptor (mGluR) (*GRM2,* log_2_FC = − 5.04; *GRM5*, log_2_FC = 5.42), adrenergic (ADRA1B, log_2_FC = − 3.49; ADRA2C, log_2_FC = − 5.36), muscarinic actylcholine (CHARM4, log_2_FC = − 2.95), opioid (OPRL1, log_2_FC = 1.53), and somatostatin receptors (SSTR5, log_2_FC = 5.30) as well as gene coding for α3 subunit of the gamma-aminobutyric acid ligand-gated receptor A (GABA_A_) (GABRA3, log_2_FC = 6.32; GABRB3, log_2_FC = 9.27) (Supplementary Fig. S8). To clarify the biological mechanisms associated with the identified Ca^2+^-related expression patterns, we focused on significantly changed GO processes related to Ca^2+^ signaling. Analysis showed 29 significantly (*P* value < 0.05) enriched GO processes, especially of Calcium ion transmembrane transport (BP), Regulation of calcium-mediated signaling (BP), Calcium channel complex (CC), and Calcium ion binding (MF) (Fig. 10D-F, Additional files 5, 6 and 7). KEGG analysis of the Ca^2+^ signaling pathway showed significant upregulation of genes related to Ca^2+^ import into the cytoplasm (*ORAI*, *CACNA1A*, *IP3R*) and significant downregulation of genes involved in Ca^2+^ export from the cytoplasm (*NCX, MCU,* and *SERCA*) in EA.hy926 cells compared to HMEC-1 cells (Supplementary Fig. S9).

## Discussion

Since its first introduction, CaEP has been tested in several clinical trials and veterinary studies on different tumor types and was found to induce a similar response as ECT with bleomycin [25, 55, 56]. Initial observations indicated that CaEP acts primarily on tumor cells, while surrounding cells are affected to a lesser degree [20]. Further studies showed different responses to CaEP even in normal cells, which were type- and tissue-dependent [22, 24, 57]. However, the effects of CaEP on the cytoskeleton, Ca^2+^ perturbations and gene expression of investigated cell types, which could drive these responses, remain largely unexplored. In this study, we investigated the effects of CaEP on two human endothelial cell lines, EA.hy926 and HMEC-1, and determined their Ca^2+^ kinetics and transcriptomic profiles. To our knowledge, this is the first in-depth study to characterize EA.hy926 and HMEC-1 cell lines to delineate the differences in responses to CaEP, Ca^2+^ kinetics and transcriptomic profiling related to cytoskeleton and Ca^2+^ signaling.

First, optimization of electroporation parameters was performed to achieve the highest permeabilization of EA.hy926 and HMEC-1 cells with minimal effect on cell survival. The obtained permeabilization profiles were similar for both cell lines (Fig. 1B). The pulse parameters that resulted in the highest permeabilization were 8 square-wave electric pulses, an amplitude to distance ratio of 1000 V/cm, a pulse length of 100 μs, and a repetition frequency of 1 Hz. This was in agreement with pulse parameters that were determined for other endothelial cell lines tested in previous studies and that are used in CaEP protocols in vivo [25, 58]. Optimized pulsed parameters were then used for CaEP of EA.hy926 and HMEC-1 cells in the presence of increasing Ca^2+^ concentrations (0 mM (control (Ctrl)), 0.5 mM, 1 mM, 2 mM and 3 mM CaCl_2_). CaEP in EA.hy926 and HMEC-1 cells in the presence of increasing Ca^2+^ concentrations showed higher cytotoxicity in EA.hy926 cells than in HMEC-1 cells (Fig. 2). The susceptibility of EA.hy926 cells to a large increase in [Ca^2+^]_i_ after CaEP exposure was further confirmed by immunofluorescence staining, which showed compromised structures of actin filaments and microtubules as well as compromised cell–cell junctions in a time- and Ca^2+^ concentration-dependent manner (Figs. 3, 4 and 5). Disruption of the plasma membrane by EP in the presence of Ca^2+^ ions was shown to cause a rapid influx of Ca^2+^ ions from the extracellular milieu due to a 10,000-fold concentration gradient across the plasma membrane, which in turn activates and recruits repair machinery for membrane resealing [59–61]. Importantly, Ca^2+^ influx not only activates and recruits the repair machinery but also drives actin cytoskeletal remodeling by depolymerizing actin filaments to G-actin and regulating microtubule disassembly [62–64], which could explain the decrease in the mean fluorescence intensity of actin filaments in EA.hy926 and HMEC-1 cells after CaEP. The higher sensitivity of EA.hy926 cells in comparison with HMEC-1 to a large increase in [Ca^2+^]_i_ after CaEP exposure may partially be due to higher expression of *CAPN2* and *CAPNS2*, genes encoding for intracellular calpain proteases which are likely to cause injury-induced cytoskeletal remodeling, as calpains have been demonstrated to be needed for Ca^2+^-dependent single-cell wound repair by cleaving downstream targets such as the intermediate filament, vimentin (*VIM*), and the actin–integrin linker, talin (*TLN1*) (Fig. 9B) [65]. Moreover, disassembly of the cortical actin cytoskeleton and microtubules is necessary, as the tensile forces generated by the cytoskeleton, which are essential for normal cell function and morphology, prevent efficient membrane repair. RNA-seq data showed significant downregulation of most investigated genes related to actin filaments (*SMARCA4*), microtubules (*TUBB*) and VE-cadherin-mediated cell–cell junctions (*CLDN5*) in EA.hy926 cells compared to HMEC-1 cells (Fig. 9C). This may explain why, despite the distinct organization of actin filaments and microtubules in EA.hy926 and HMEC-1 monolayers before treatment, the lower mean fluorescence intensities of individual cytoskeletal structures in EA.hy926 cells after CaEP suggest more pronounced morphological changes and a diminished cytoskeletal repair ability compared to HMEC-1 cells. However, additional protein-based assays are necessary to further support these observations. Actin filaments, microtubules and cell-cell junctions were all affected by CaEP, especially at 2 and 3 mM Ca^2+^ concentrations. However, according to immunofluorescence images and mean fluorescence intensity of VE-cadherin cell-cell junctions maintained their structural integrity. This is consistent with observed Ca^2+^-dependent membrane repair after electropermeabilization, where perfusion with 2 mM external Ca^2+^ advanced membrane resealing, which could have a positive effect on cell-cell junction preservation [66]. Although immunofluorescence of VE-cadherin 1 h after treatment shows that both cell lines are similarly affected, the higher mean fluorescence intensity of VE-cadherin in HMEC-1 cells at 4 and 24 h time points may indicate more pronounced Ca^2+^-mediated membrane resealing compared to Ea.hy926 cells (Fig. 5B). As in the case of mean fluorescence intensity, the maximum fluorescence intensity highlighted noticeable differences between EA.hy926 and HMEC-1 cells, however the ratios of fluorescence signals of VE-cadherin on the plasma membrane (PM) to the VE-cadherin fluorescence signals of the cytosol (CS) were not significantly affected (Supplementary Fig. S5).

To determine whether differences in the sensitivity of EA.hy926 and HMEC-1 cells in response to CaEP and cytoskeletal reorganization might be related to the expression of specific Ca^2+^-related ion channels and receptors, fluorometric kinetic measurements of [Ca^2+^]_i_ were performed on EA.hy926 and HMEC-1 cells in response to mechanical stress, Ca^2+^ influx from the extracellular space and ligand-mediated mobilization of intracellular stores (Fig. 6A). The exposure of EA.hy926 and HMEC-1 cells to mechanical stress induced by injection of buffer (DPBS) induced a minimal response, but the response was significantly higher in EA.hy926 cells, which suggested that EA.hy926 cells have higher expression of mechanosensing ion channels than HMEC-1 cells (Fig. 6B). The differences in response to shear stress in previous studies were attributed to different levels of expression of *CDH5*, *VEGFR2*, *PIEZO1/2 CCM1/2/3* and *ITGBP1*, as observed in HUVECs and EA.hy926 cells [27, 67]. In our study, DGE analysis of EA.hy926 and HMEC-1 cells showed significant differences in the expression of mechanoreceptor *TRPV* genes as well as *PIEZO1/2* and *PKD1/2*. Interestingly, *TRPVs*, *PIEZO1/2* and *PKD1* were all downregulated in EA.hy926 cells compared with HMEC-1 cells, suggesting the involvement of other mechanoreceptors in response to DPBS-induced shear stress. Exposure to ionomycin showed a comparable time pattern of Ca^2+^ influx between the cell lines; however, there was a significantly higher maximum [Ca^2+^]_i_ amplitude response in HMEC-1 cells (Fig. 6C). In contrast, specific depletion of Ca^2+^ stores with thapsigargin via inhibition of SERCA pumps resulted in faster Ca^2+^ depletion in EA.hy926 cells but no significant difference in the maximum [Ca^2+^]_i_ amplitude response compared with HMEC-1 cells (Fig. 6A and D), which suggests that EA.hy926 and HMEC-1 cells have similar stores of intracellular Ca^2+^. The variation in kinetics may be attributed to the upregulation of IP3R and the downregulation of SERCA in EA.hy926 cells (Supplementary Fig. S9). GPCR-dependent responses were induced by selective agonists of G_αq/11_-coupled metabotropic P2Y, bradykinin, angiotensin II, and muscarinic acetylcholine receptors, which activate phospholipase C, resulting in the release of IP_3_ and subsequent mobilization of intracellular Ca^2+^ from the ER Ca^2+^ store [68]. Among them, ATP induced the highest increase in [Ca^2+^]_i_, which was comparable in both cell lines in regard to time-course and maximum amplitude response (Fig. 6A and E). ATP is known to bind both to G protein-coupled P2Y receptors and ionotropic P2X receptors [69]. In the absence of Ca^2+^ in the external medium, ATP-induced changes in [Ca^2+^]_i_ were reduced by approximately 80% in EA.hy926 cells and virtually absent in HMEC-1 cells (Fig. 7), indicating that ATP-induced Ca^2+^ increases in HMEC-1 cells are mainly due to Ca^2+^ entry through ionotropic P2X receptors. On contrary, findings in EA.hy926 cells suggest that in addition to the involvement of ionotropic P2X receptors, a part of the increase in [Ca^2+^]_i_ can be attributed to the activation of metabotropic P2Y receptors that trigger Ca^2+^ release from the intracellular ER store. This was in line with RNA-seq results, which showed significantly increased expression of genes encoding for P2Y receptor subtypes (*P2RY6*) in EA.hy926 cells and similar expression of P2X receptor subtypes (*P2RX5* and *P2RX7* were downregulated, while *P2RX6* was upregulated) compared with HMEC-1 cells (Fig. 10B). Changes in [Ca^2+^]_i_ induced by bradykinin, acetylcholine and angiotensin II were substantially lower than those induced by ATP (Fig. 6A, F-H). Among them, bradykinin induced the highest maximum amplitude response in [Ca^2+^]_i_. Both bradykinin and angiotensin II induced significantly higher [Ca^2+^]_i_ in EA.hy926 cells than in HMEC-1 cells (Fig. 6A, F and H). The same trend was observed for an otherwise very low response to acetylcholine (Fig. 6A and G). In our previous studies involving neuroblastoma NG108–15 and vascular smooth muscle A10 cell lines, we conducted [Ca2+]i measurements on both individual adherent cells and cell populations (trypsinized cells) [42, 43]. Individual adherent cells were assessed using an inverted Leica multispectral laser scanning confocal microscope, while for cell populations we utilized the TriStar LB 940 multimode microplate reader, as in the present study. Given that the TriStar LB 940 is equipped with injectors and has precise temperature control, we believe that time-dependent responses are more accurate when measured on cell populations. It is important to note a limitation of our study, as we did not conduct [Ca^2+^]_i_ kinetic experiments on adherent cells but only on cell populations, which may limit our ability to fully understand calcium dynamics in adherent cells.

Global transcriptomic profiling of untreated EA.hy926 and HMEC-1 cells showed 5366 significant DEGs between the cell lines involved in 149 significantly overrepresented signaling pathways according to KEGG analysis. Categorization of DEGs between EA.hy926 and HMEC-1 cell lines into three enriched categories based on gene ontology analysis (BP, CC, MF) showed significantly enriched processes related to the regulation of cellular and molecular processes, metabolism, development, membrane, intracellular organelles and protein binding (Fig. 8). These results were expected, as the two studied endothelial cell lines differ in their tissue origin and derivation method.

Differences in F-actin, β-tubulin, and VE-cadherin observed after CaEP by immunofluorescence staining were investigated by comparing the cytoskeleton-related transcriptomic profiles of EA.hy926 and HMEC-1 cell lines (Fig. 9). The most morphological differences in cytoskeletal components between untreated cell lines were observed in actin filaments. Out of 55 analyzed cytoskeleton-related genes, *ICAM2, MYH3* and *PECAM1* were the top three significantly upregulated in EA.hy926 cells compared to HMEC-1 cells (Fig. 9B). In contrast, *CLDN5*, *NEXN* and *NFATC-1* were found to be the top three significantly downregulated genes in EA.hy926 cells compared to HMEC-1 cells (Fig. 9B). GO analysis showed significantly enriched GO processes related to the cytoskeleton, especially in actin-related processes and adhesion molecule binding (Fig. 9C-E). Additionally, KEGG analysis revealed significant enrichment of the regulation of the actin cytoskeleton (Fig. 9B). While upregulated genes in EA.hy926 did not show a protective effect against CaEP, upregulation of the actin filament-related genes *NEXN* and *ANLN* in HMEC-1 cells compared to EA.hy926 cells may be responsible for the organization of actin filaments to extend throughout the cell body and cytoskeleton protection after CaEP.

To further determine differences in [Ca^2+^]_i_ between EA.hy926 and HMEC-1 cells after exposure to agonists of Ca^2+^ channels, specific genes and pathways related to Ca^2+^ signaling and transport through different cellular compartments were investigated. Among the 72 analyzed Ca^2+^signaling-related genes, *TRPM6*, *CACNG7*, and *TRPM2* were the top three upregulated, while *TRPV2, TRPV4* and *PIEZO2* were the top three significantly downregulated Ca^2+^ signaling-related genes in the EA.hy926 cell line compared to the HMEC-1 cell line (Fig. 10B). When looking at genes involved in Ca^2+^ influx, the EA.hy926 cell line showed significantly higher expression of the *ORAI2*, *TRPC1*, *TRPM2*, CNGA3 and *TRPM6* genes and significantly lower expression of *TRPV4* and *TRPC4* than the HMEC-1 cell line (Fig. 10C). Genes involved in Ca^2+^ efflux were similarly expressed in both cell lines (Fig. 10D). Analysis of GO processes related to Ca^2+^ signaling showed 29 significantly enriched GO processes, including calcium ion transmembrane transport, regulation of calcium-mediated signaling, calcium channel complex, and calcium ion binding (Fig. 10D-F). Moreover, KEGG analysis of the Ca^2+^ signaling pathway showed significant upregulation of genes related to Ca^2+^ import into the cytoplasm (*ORAI*, *CACNA1A*, *IP3R*) and significant downregulation of genes involved in Ca^2+^ export from the cytoplasm (*NCX, MCU,* and *SERCA*) in EA.hy926 cells compared to HMEC-1 cells (Supplementary Fig. S9). Taken together, the distinct transcriptomic profile of EA.hy926 cells with upregulated expression of Ca^2+^ influx genes and downregulation of Ca^2+^ efflux genes could be crucial for the sensitivity of EA.hy926 cells to Ca^2+^ regulation, which could explain the detrimental effects of CaEP as well as the higher maximum [Ca^2+^]_i_ amplitude responses in kinetic experiments compared with HMEC-1 cells. However, further research on the protein level is needed.

Increased [Ca^2+^]_i_ potentially triggers rapid and/or sustained events, ranging in seconds/minutes and hours/days, respectively. Although the described data provide evidence that these responses are more prominent in EA.hy926 cells than in HMEC-1 cells, single-cell analysis points to the existence of variability in the same cell population, depending on the quali-quantitative differential expression of receptors, signaling molecules, calcium channels and other elements of intracellular signaling [70]. Nonetheless, our observations suggest that there are distinct differences between the human endothelial cell lines EA.hy926 and HMEC-1, which could determine the suitability of models for specific studies related to ECs. Several independent lines of evidence suggest a critical role for Ca^2+^ in the control of angiogenesis and tumor progression: in particular, Ca^2+^ entry from the extracellular space, mediated by the opening of plasma membrane Ca^2+^-permeable channels, is involved in the control of proliferation in virtually all cell types [71, 72]. For these reasons, the Ca^2+^ signaling cascade represents a potential target for the development of anticancer drugs using natural or synthetic Ca^2+^ channel blockers [72, 73]. However, when evaluating such anticancer drugs, their effect on nontumor cells such as EA.hy926 and HMEC-1 cells needs to be considered, while the cell line model should be selected depending on the expression of particular Ca^2+^-related channels in question. Moreover, EA.hy926 sensitivity to shear stress could be exploited, particularly in the case of atherosclerosis. Namely, in atherosclerosis, ECs are longitudinally aligned in the direction of flow, while in low shear stress regions, ECs are poorly aligned [74]. Therefore, EA.hy926-based assays could provide an insightful link between low wall shear stress and atherogenesis, which has long been the subject of investigation but has yet to be fully elucidated.

To summarize, this study demonstrates the effects of CaEP on the native endothelium of blood vessels represented by two human endothelial cell lines, EA.hy926 and HMEC-1. EA.hy926 and HMEC-1 cells morphologically differ and show different responses to CaEP and specific agonists of Ca^2+^ channels and receptors. Compared to HMEC-1 cells, EA.hy926 cells are more sensitive to CaEP and to changes in [Ca^2+^]_i_, which could be due to the distinct transcriptomic profile of cytoskeletal, Ca^2+^ influx and Ca^2+^ efflux genes. In addition, our study provides a bioinformatic basis for the selection of the EC model depending on the objective of the research.

### Supplementary Information

**Additional file 1.**

**Additional file 2.**

**Additional file 3.**

**Additional file 4.**

**Additional file 5.**

**Additional file 6.**

**Additional file 7.**

**Additional file 8.**

## Data Availability

The data that support the findings of this study are available from the corresponding authors upon reasonable request. RNA sequencing data obtained and discussed in this publication have been deposited in the NCBI Gene Expression Omnibus (GEO) and are accessible through GEO Series accession number GSE244042 (https://www.ncbi.nlm.nih.gov/geo/query/acc.cgi?acc=GSE244042).

## Abbreviations

ECs: endothelial cells
CaEP: calcium electroporation
[Ca^2+^]_i_: intracellular free Ca^2+^ concentration
GPCR: G protein coupled receptor
DEGs: differentially expressed genes
DGE: differential gene expression
ER: endoplasmic reticulum
EP: electroporation
ECT: electrochemotherapy
RNA-seq: RNA sequencing
PPIs: clusters of human protein–protein interactions
DPBS: Dulbecco’s phosphate-buffered saline
EPB: electroporation buffer
CSB: cytoskeleton stabilizing buffer
HBSS^+/+^: Hank’s Balanced Salt Solution supplemented with Ca^2+^ and Mg^2+^
