# Supplementary material for: Characterization of two distinct immortalized endothelial cell lines, EA.hy926 and HMEC-1, for in vitro studies: exploring the impact of calcium electroporation, Ca2+ signaling and transcriptomic profiles
Source: Cell Commun Signal. 2024 Feb 12;22:118. doi: 10.1186/s12964-024-01503-2 (PMC10863159; doi:10.1186/s12964-024-01503-2)
Supplement: Supplementary file 1 — Additional file 1. [file 12964_2024_1503_MOESM1_ESM.docx]

**
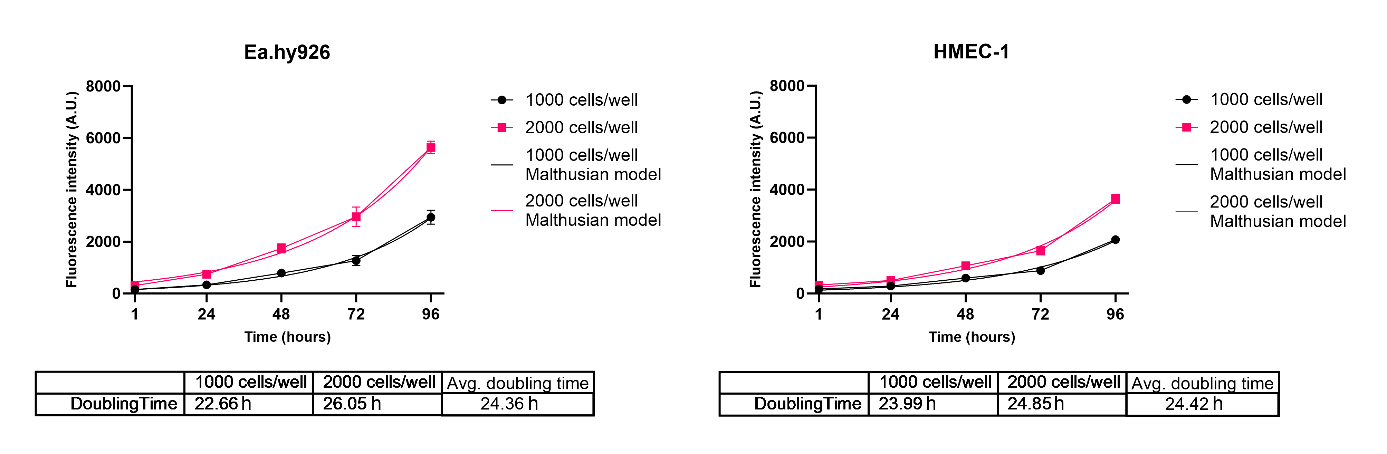
**

**Supplementary Fig. S1.** Growth curves of Ea.hy926 and HMEC-1 cells. The cells were seeded at densities of 1000 and 2000 cells per well on a 96-well plate. Cell viability was assessed at various time points (1, 24, 48, 72, and 96 h) using the PrestoBlue assay and measured with the Cytation 1 imaging system. The doubling times for each cell line were determined by fitting the data to the Exponential (Malthusian) growth curve model.


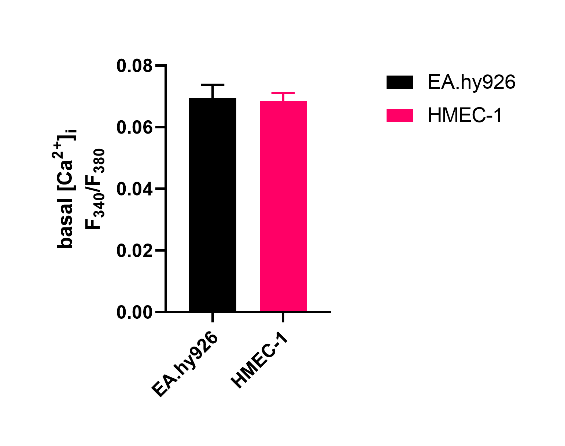


**Supplementary Fig. S2.** Basal levels of [Ca^2+^]_i_ of Ea.hy926 and HMEC-1 cells. Baseline fluorescence obtained in the initial measurement period of 40 s, i.e., before the injection of the test substance, was used to assess basal [Ca^2+^]_i_ in both cell lines. Averaged F_340_/F_380_ ratios obtained before the application of the test substance, reflecting basal [Ca^2+^]_i_, were comparable between both cell lines.
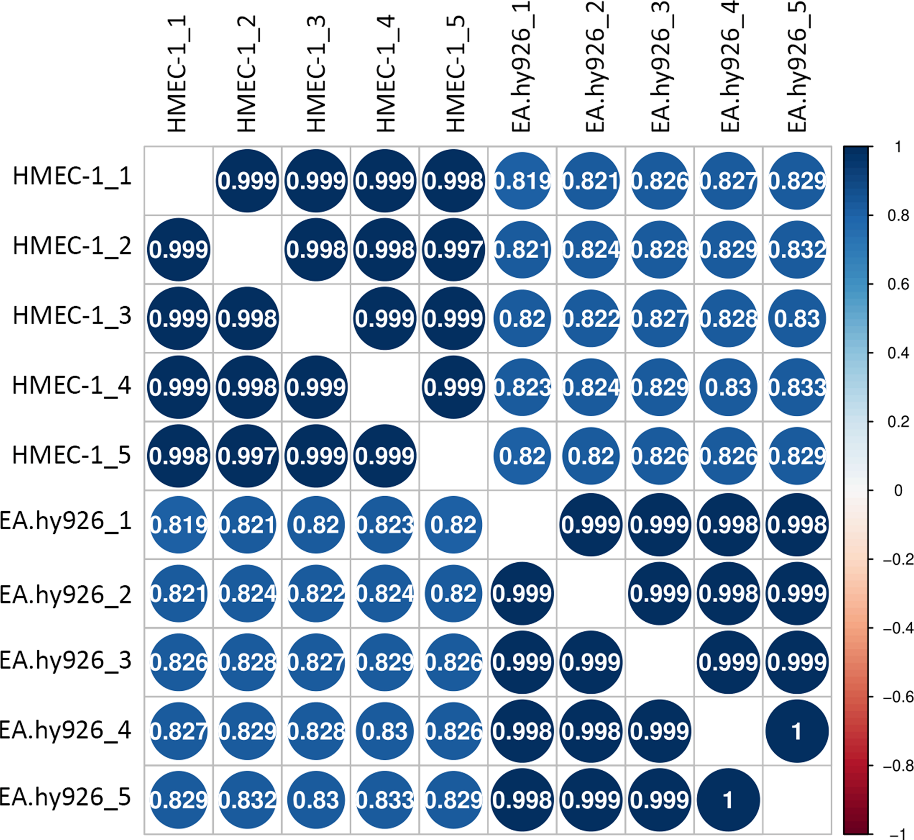


**Supplementary Fig. S3.** Pearson correlation between samples of EA.hy926 and HMEC-1 cells. Pearson’s correlation plot visualizing the correlation values between samples. Scale bar represents the range of the correlation coefficients displayed.


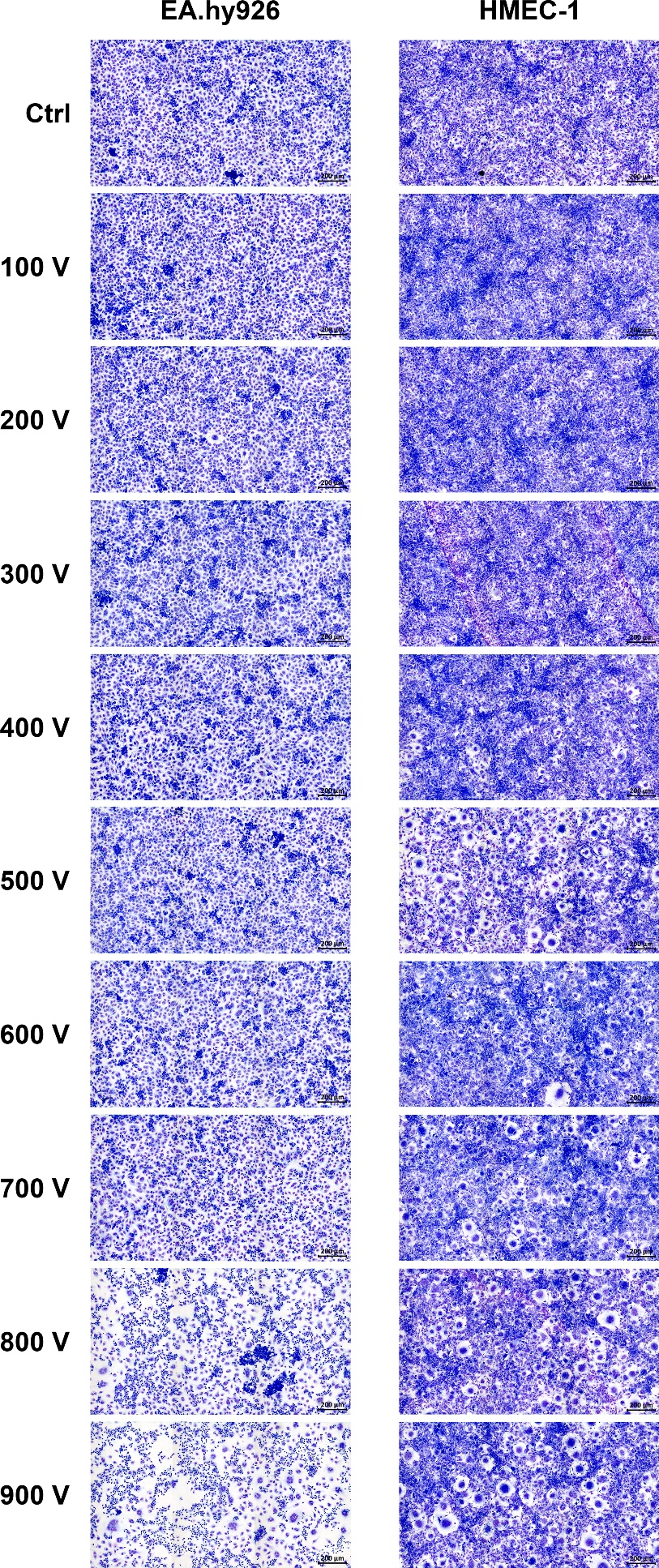


**Supplementary Fig. S4.** Endothelial cells EA.hy926 and HMEC-1 after electroporation. Representative images show untreated monolayer of EA.hy926 and HMEC-1 cells after exposure to electric pulses with increasing voltage stained with Giemsa stain. Scale bar: 200 µm.


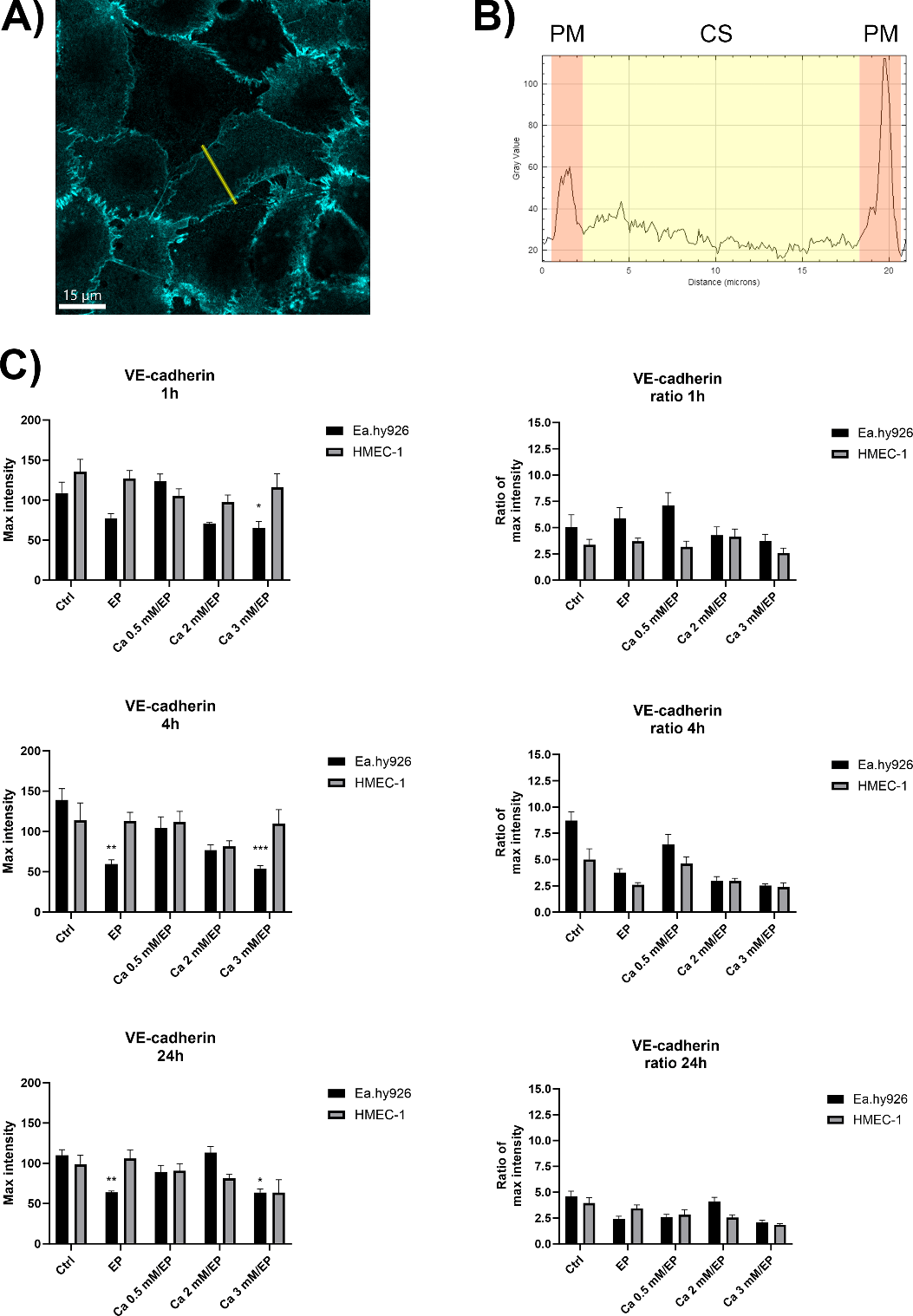


**Supplementary Fig. S5.** Maximum fluorescence intensity of cell‒cell junctions in EA.hy926 and HMEC-1 cells. A) Representative confocal image of VE-cadherin in control Ea.hy926 cell monolayer at 1 h time point and B) a typical line scan indicated by the dotted line in cell. Scale bar: 15 µm, line width: 20 px, PM: plasma membrane, CS: cytosol. C) Maximum fluorescence intensity of VE-cadherin and the average ratios (PM/CS) of the fluorescence signals of the VE-cadherin on the plasma membrane (PM) to the VE-cadherin fluorescence signals of the cytosol (CS) of representative cells are indicated as AM ± SEM (n ≥ 3). Statistical significance was determined using a nonparametric one-way ANOVA (Kruskal-Wallis test). A P value of < 0.05 was considered to be statistically significant (*P < 0.05, ** P < 0.01, and *** P < 0.001 vs control (Ctrl)).


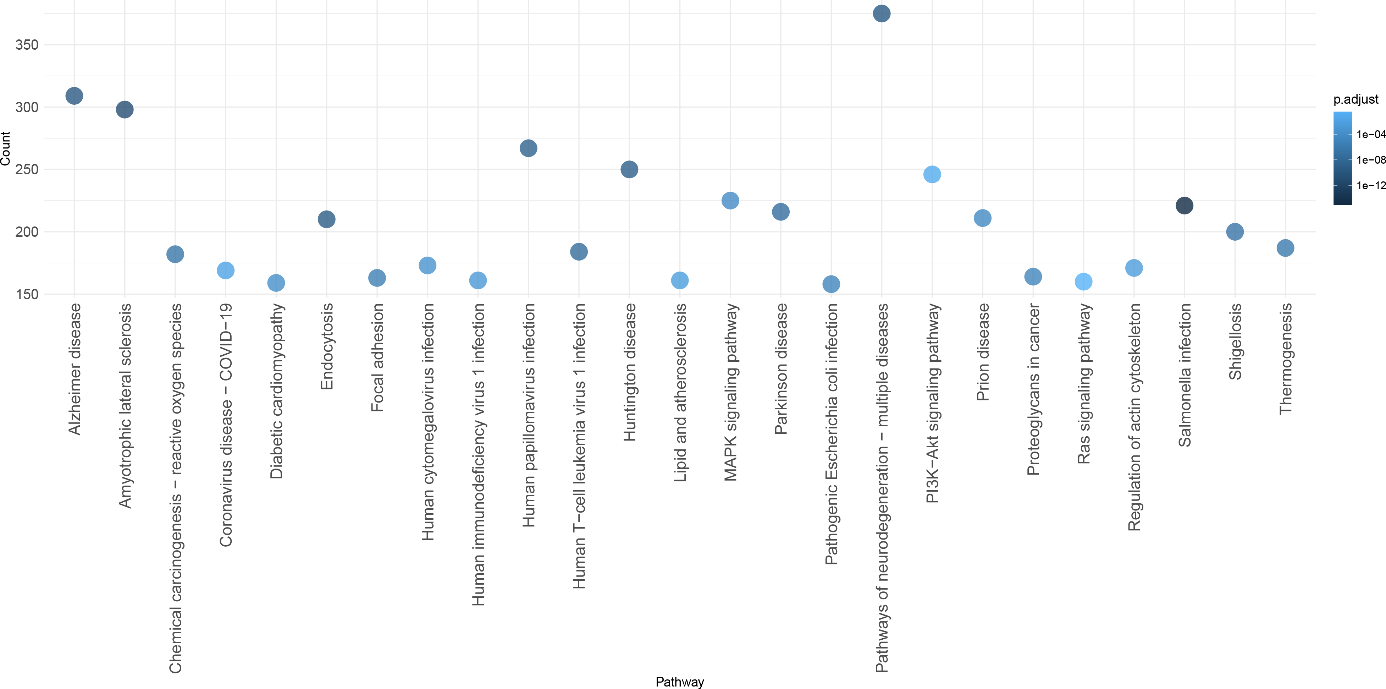


**Supplementary Fig. S6.** Dot plot showing the top 25 significantly enriched KEGG pathways based on the number of DEGs (Count) involved. An adjusted P value of < 0.05 was considered statistically significant.


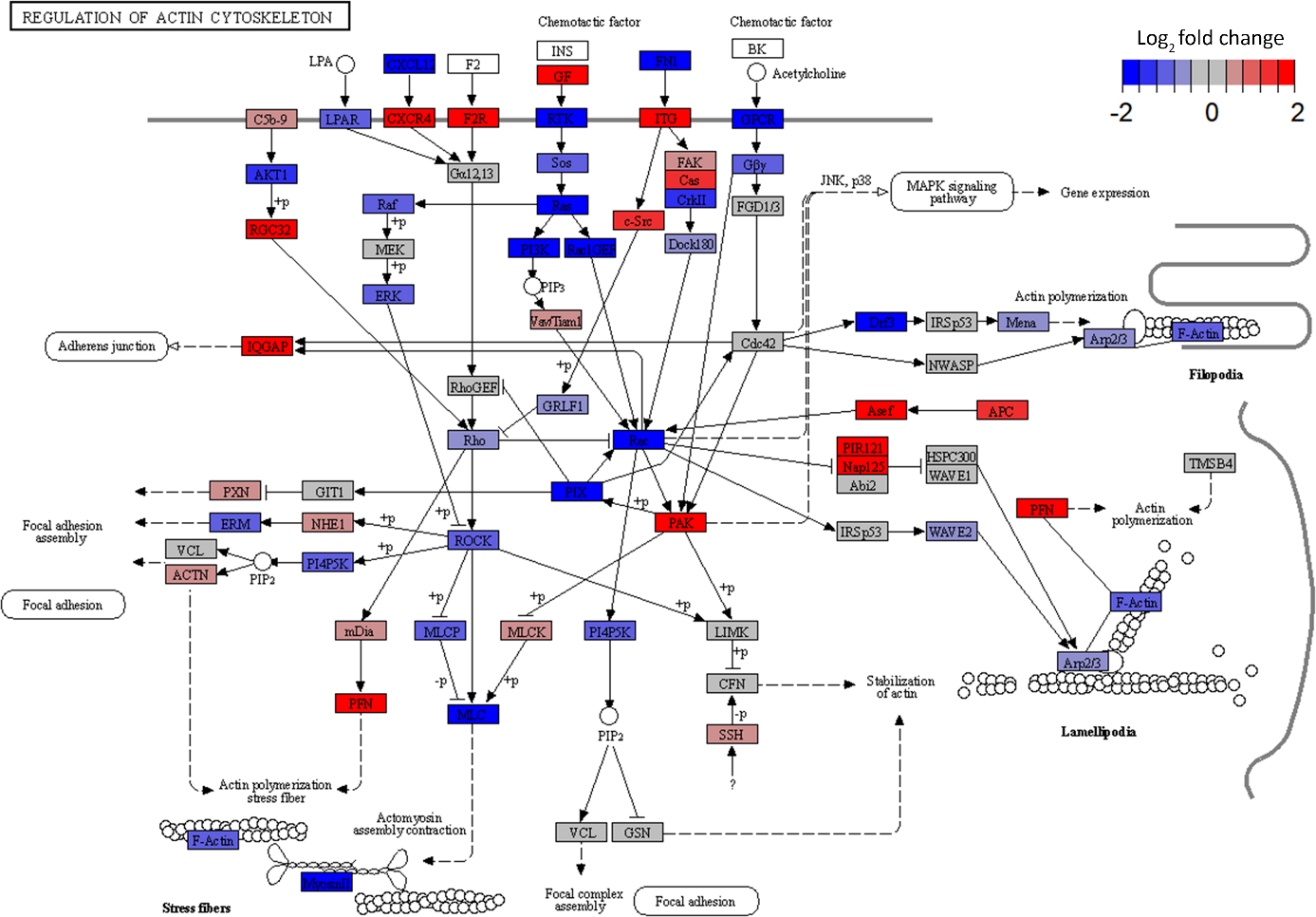


**Supplementary Fig. S7.** KEGG analysis of Regulation of actin cytoskeleton (hsa04810) presented by usage of the *Pathview* R package. Genes are colored based on log_2_FC. Genes in white rectangles were not significantly expressed (P value > 0.05).


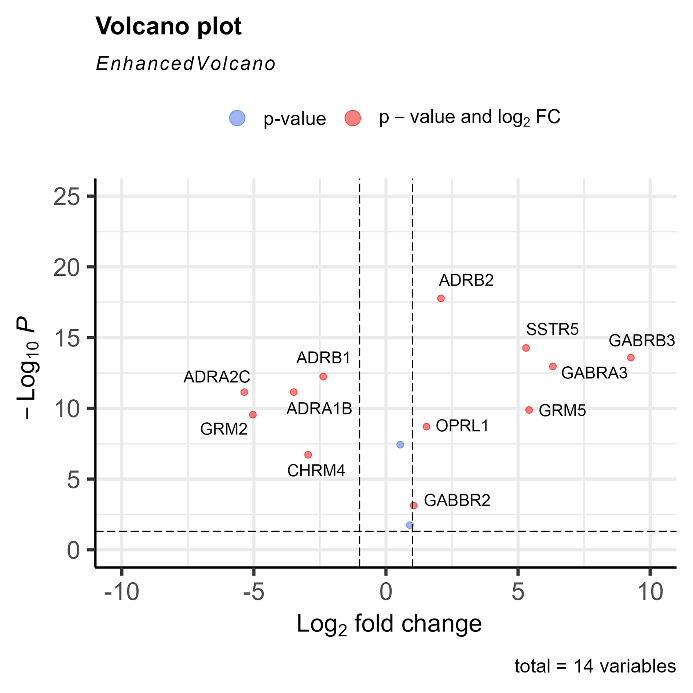


**Supplementary Fig. S8.** Volcano plot showing additional differentially expressed GPCR receptors related to Ca^2+^ signaling to the cytoskeleton in EA.hy926 and HMEC-1 cells. Genes with adjusted P values of < 0.05 and |log_2_FC| > 1 were considered to be statistically significant. A total of 14 genes were analyzed. FC - fold change.


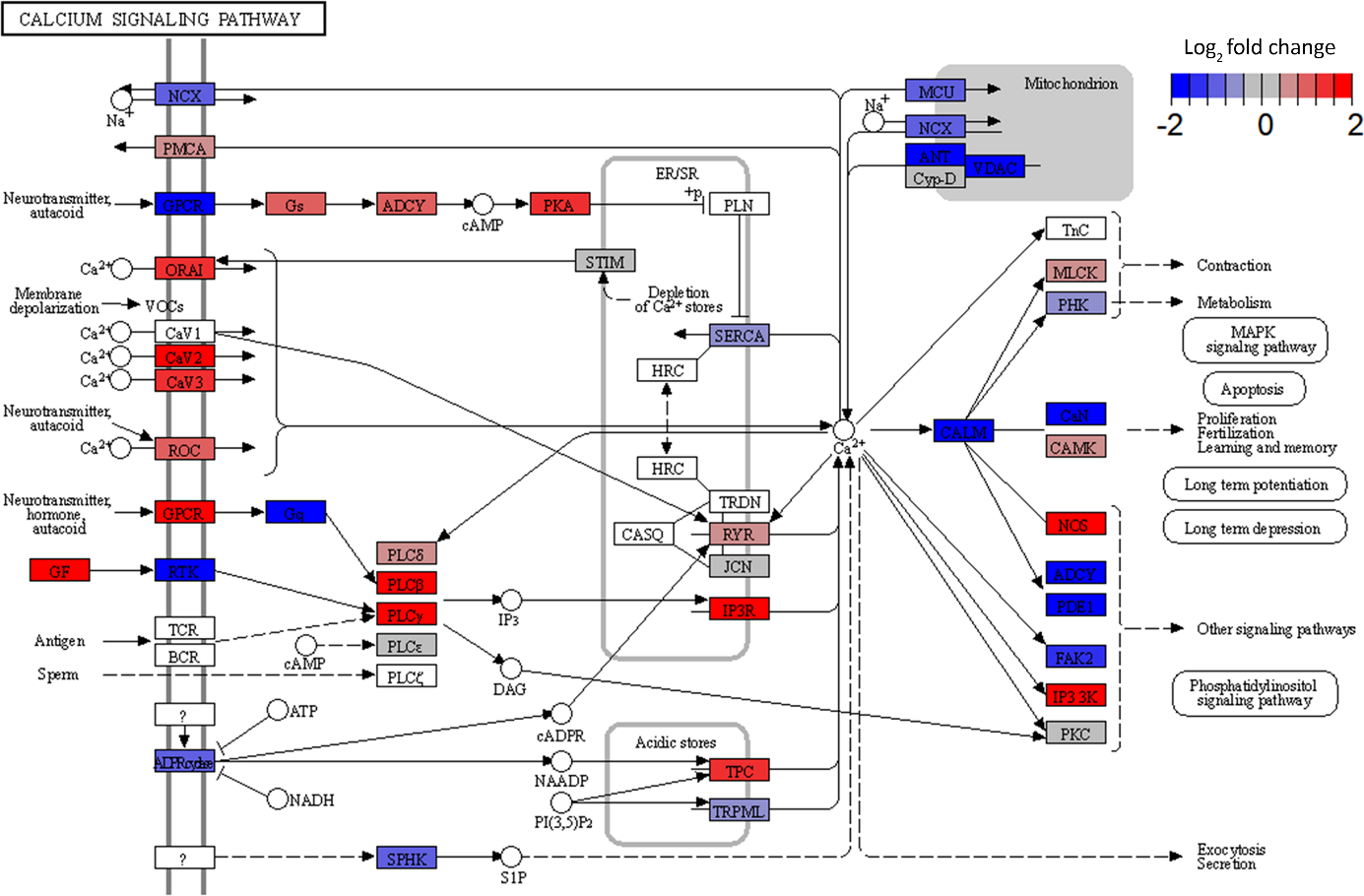


**Supplementary Fig. S9.** KEGG analysis of the Ca^2+^ signaling pathway (hsa04020) presented by usage of the *Pathview* R package. Genes are colored based on log_2_FC. Genes in white rectangles were not significantly expressed (P value > 0.05).
